# Genetic Analysis of Heterosis for Yield Influencing Traits in *Brassica juncea* Using a Doubled Haploid Population and Its Backcross Progenies

**DOI:** 10.3389/fpls.2021.721631

**Published:** 2021-09-16

**Authors:** Aakanksha, Satish Kumar Yadava, Bal Govind Yadav, Vibha Gupta, Arundhati Mukhopadhyay, Deepak Pental, Akshay K. Pradhan

**Affiliations:** ^1^Department of Genetics, University of Delhi South Campus, New Delhi, India; ^2^Centre for Genetic Manipulation of Crop Plants, University of Delhi South Campus, New Delhi, India

**Keywords:** *Brassica juncea*, heterosis, QTL mapping, yield influencing traits, dominance, epistasis

## Abstract

The exploitation of heterosis through hybrid breeding is one of the major breeding objectives for productivity increase in crop plants. This research analyzes the genetic basis of heterosis in *Brassica juncea* by using a doubled haploid (DH) mapping population derived from F_1_ between two heterotic inbred parents, one belonging to the Indian and the other belonging to the east European gene pool, and their two corresponding sets of backcross hybrids. An Illumina Infinium Brassica 90K SNP array-based genetic map was used to identify yield influencing quantitative trait loci (QTL) related to plant architecture, flowering, and silique- and seed-related traits using five different data sets from multiple trials, allowing the estimation of additive and dominance effects, as well as digenic epistatic interactions. In total, 695 additive QTL were detected for the 14 traits in the three trials using five data sets, with overdominance observed to be the predominant type of effect in determining the expression of heterotic QTL. The results indicated that the design in the present study was efficient for identifying common QTL across multiple trials and populations, which constitute a valuable resource for marker-assisted selection and further research. In addition, a total of 637 epistatic loci were identified, and it was concluded that epistasis among loci without detectable main effects plays an important role in controlling heterosis in yield of *B. juncea*.

## Introduction

Heterosis is defined as the superior performance of F_1_ hybrids relative to the homozygous parents (East, 1908; Shull, 1908). While the practical application of heterosis in plant breeding is quite successful in many crops through the development of hybrid varieties, the molecular mechanism of the phenomenon is not well understood. The development of molecular quantitative genetics has facilitated the study of the genetic basis of heterosis in crops (Paterson et al., 1988; Stuber, 1992). Numerous studies have been carried out in different crops, including maize, which is an outcrossing crop, and rice and wheat, which are self-pollinated. Three main hypotheses exist to explain the genetic basis of heterosis—dominance, overdominance, and epistasis (Crow, 1999; Goodnight, 1999; Lippman and Zamir, 2007). The dominance hypothesis supposes that deleterious recessive alleles of one of the parents are complemented in the F_1_ hybrid by the dominant alleles of the other parent. Results from several quantitative genetic experiments in crops like rice and maize favor the dominance hypothesis (Xiao et al., 1995; Cockerham and Zeng, 1996; Swanson-Wagner et al., 2009). The overdominance hypothesis states that the heterozygous combination of the alleles at a locus is superior to either of the two possible homozygous combinations. Overdominance has been seen as the primary genetic basis of heterosis in different crops like maize (Stuber et al., 1992), rice (Li et al., 2001; Luo et al., 2001; Mei et al., 2005; Zhu et al., 2016), tomato (Semel et al., 2006; Krieger et al., 2010), and cotton (Ma et al., 2019). Epistasis is defined as the interactions between alleles of different loci and has been shown as the major determinant of heterosis in a few studies (Yu et al., 1997; Hua et al., 2002, 2003; Luo et al., 2009; Tang et al., 2010; Zhou et al., 2012).

In *Brassica napus*, Radoev et al. (2008) used doubled haploids (DH) and the corresponding backcross lines with their midparent heterosis data to map heterotic quantitative trait loci (QTL) and identified a large number of epistatic interactions for seed yield and three yield-component traits. They concluded that epistasis, together with all levels of dominance from partial to overdominance, was responsible for the expression of heterosis in rapeseed. Using the same experimental design in two DH populations and two corresponding sets of backcross hybrids, Basunanda et al. (2010) detected a number of QTL hotspots, which consisted of epistatic loci as well as main-effect QTL associated with seedling biomass and yield-related traits. They found that epistatic interactions played an important role in the expression of heterosis in oilseed rape and postulated that these QTL hotspots might harbor genes involved in the regulation of heterosis for different traits, with an overall significant influence on heterosis for seed yield. Prominent clustering of QTL for yield and yield-related traits have also been reported in genomes of many other crops and model plants, such as rice (You et al., 2006), maize (Frascaroli et al., 2007), wheat (Peng et al., 2003), mustard (Ramchiary et al., 2007; Yadava et al., 2012), and Arabidopsis (Fu et al., 2009), indicating that QTL for yield-related traits might have pleiotropic effects. Studies on various crop species have consistently postulated that the genetic mechanism of heterosis is complex without any single explanation for its expression (Radoev et al., 2008; Liang et al., 2015; Shang et al., 2016; Li et al., 2018; Ma et al., 2019; Liu et al., 2020).

Different mapping populations with varied genetic structures have previously been used to study heterosis in crops (Schnable and Springer, 2013). F_2_ and BC populations can be used to study heterosis; however, their temporary nature in the form of single plants as the segregating units restricts their use in experiments with replications (Yu et al., 1997; Li et al., 2012). To circumvent these issues, new population types in the form of “immortalized F2” (IF2) populations and double backcrosses of DH or Recombinant inbred lines (RILs) have been constructed in several studies (Xiao et al., 1995; Hua et al., 2002, 2003; Mei et al., 2005; Shi et al., 2011). Several studies on heterosis using a backcross design have been carried out in different crops, such as rice (Xiao et al., 1995; Li et al., 2001; Luo et al., 2009), maize (Frascaroli et al., 2007; Larièpe et al., 2012), and rapeseed (Radoev et al., 2008; Basunanda et al., 2010).

Oilseed mustard, *Brassica juncea* (AABB), an important oilseed crop of the Indian subcontinent is grown under low-moisture regimes during the winter-growing season. The major breeding objectives in *B. juncea* target yield enhancement, oil quality, resistance against pathogens, and tolerance for abiotic stresses (Pradhan and Pental, 2011). Extensive phenotypic and DNA marker-based studies on oilseed types of *B. juncea* have confirmed the existence of two major distinct gene pools—-Indian and East European (Pradhan et al., 1993; Srivastava et al., 2001). Hybrids between the Indian and east European types are heterotic for yield (Pradhan et al., 1993). In *B. juncea*, the first ever commercial hybrid DMH-1 released in India, developed from a cross between an Indian gene pool line Pusa bold and an east European gene pool line EH-2, shows high heterosis with about 30% higher yield than the best check varieties (Sodhi et al., 2006). The molecular basis of heterosis in *B. juncea* has not been investigated so far, and there remain several unresolved basic questions on the genetics of this highly complex trait. The principal objective of this study was, therefore, the genetic analysis of heterosis by identification, localization, and estimation of the effects of QTL for seed yield and related component traits, along with an exhaustive assessment of the contributions of different genetic effects, e.g., dominance, overdominance, and epistasis, to the expression of heterosis in *B. juncea*.

## Materials and Methods

### Plant Materials

A DH mapping population was derived from a cross between an archetypical Indian gene pool line of mustard—Varuna, and EH-2, an early flowering mutant of Heera (the canola-quality exotic eastern European line). The two lines are highly divergent and good heterotic combiners belonging to two distinct gene pools—Indian and east European. Briefly, crosses were made between Varuna and EH-2, using Varuna as the female parent. F_1_ plants were grown in controlled environmental chambers for the isolation of microspores to obtain DH plants through *in vitro* colchicine treatment of microspores and embryogenesis (Mukhopadhyay et al., 2007). Second or third leaf from 3-to-4-week-old individual putative doubled haploid plants was taken for analyzing its ploidy level, following Arumuganathan and Earle (1991), and the diploidized plants were transplanted in the field.

The DH population (hereinafter referred to as “VEH”) consisted of 164 lines developed from the F_1_ of a cross between Varuna and EH-2. Two backcross (BC) populations were developed from crosses between the DH lines with Varuna and with EH-2 by using the DH lines as the female parent, and Varuna or EH-2 as the male parent, hereinafter referred to as “BC-V” and “BC-E,” respectively. Each of these two BC populations consisted of 164 BCF_1_ hybrids corresponding to the 164 DH lines of the VEH population.

### Field Experiments and Data Analysis

The DH lines, the backcross populations, the two parental lines and the F_1_ of the Varuna/EH-2 cross were grown in fields located in Delhi during the crop seasons of 2014 to 2015, 2015 to 2016, and 2016 to 2017 (Supplementary Table 1), following a randomized complete block design with three replications in each of the three trials. Phenotyping for yield-influencing traits was performed with five competitive plants from each replication, and the mean of the 15 observations was used as the trait value. The traits were broadly classified as plant architectural traits and days to flowering, silique-related traits, and seed-related traits as described in literature (Cai et al., 2016; Zhao et al., 2019, 2021). A total of 14 characters defining plant architecture, days to flowering, silique-related traits, and seed-related traits were evaluated in the three mapping populations: (1) plant height (PH); (2) days to 50% flowering (DF); (3) number of primary branches (PBR); (4) number of secondary branches (SBR); (5) main shoot length (MSL); (6) single plant yield (SPY); (7) silique on the main shoot (SQMS); (8) silique length (SQL); (9) seeds in a silique (SSQ); (10) silique density (SQD); (11) number of siliques on a plant (SQPL); (12) thousand seed weight (TSW); (13) oil content (OIL); and (14) protein content (PRO).

The levels of midparent heterosis were estimated for the F_1_ hybrid of the parents “Varuna” and “EH-2,” referred to as F_1_-heterosis. For testing the significance of heterosis values, *t-*tests were applied. The data were statistically analyzed using Plabstat (Utz, 2004), and the trait ANOVAs were performed using the phenotyping module of iMAS (Integrated Marker Assisted Selection) version 2.1 (http://www.icrisat.org/gt-bt/imas.htm). The adjusted mean (Best Linear Unbiased Prediction, BLUP) values across the three environments were calculated with a mixed linear model using the R package metan (Olivoto and Lúcio, 2020), accounting for effects of environment, replication, genotype, and genotype by the environment.

### Genetic Mapping

Young leaves were collected from the parents, F_1_ and DH lines, and stored at −80°C. Total genomic DNA was isolated from the leaves, following the CTAB method (Rogers and Bendich, 1994). Polymorphic markers available in the lab were used for genotyping the VEH DH mapping population and were used to develop the framework linkage map. Two types of agarose gel-based markers —Intron Polymorphism (IP) (Panjabi et al., 2008) and simple sequence repeat (SSR) (Dhaka et al., 2017)—and a third type of markers—SNP—identified by PCR-based KASP assays, which were previously developed using the transcriptome data for two *B. juncea* varieties —Varuna and Heera (Paritosh et al., 2014)—were used in this study. These IP, SSR, and KASP-SNP markers served as anchor markers, which were used to identify the linkage groups and their positions on the VEH linkage map corresponding to their previously known positions on the 10 A sub-genome and 8 B sub-genome linkage groups of the *B. juncea* linkage map, following Paritosh et al. (2014).

For the development of an SNP-based high-density genetic map of *B. juncea*, Illumina Infinium Brassica 90K SNP array was used as it could generate large amount of marker data in a single experiment with reasonably good distribution across the genome and high-information content, including physical positions. The availability of a large number of markers genotyped on the VEH population entailed genetic mapping in the R statistical computing environment using the ASMap package (Taylor and Butler, 2017; R Core Team, 2020). Genotypes of the backcross hybrids constituting the two BC populations were inferred from the genotypes of the VEH lines.

### Correlation Analysis

Phenotypic data were organized into five data sets: DH (VEH), two backcrosses (BC-V and BC-E), and two corresponding midparent heterosis data sets (MPH-V and MPH-E). Correlation coefficients between trait values in five data sets, BLUPs, and those between genome heterozygosity and trait performance were estimated in the R statistical environment (R Core Team, 2020). Mean of the trait values of three replications of each of the data set in individual trials and BLUPs for the combined analysis were used to calculate correlation coefficients between five data sets.

To calculate correlation coefficients between genome heterozygosity and trait performance in the BC populations, genome heterozygosity for the two BC populations was assessed from the genome ratios of the 164 doubled haploid lines. Genome heterozygosity for the BC-V (VEH × Varuna) population was calculated as the percentage of genome, which originated from EH-2, and that for BC-E (VEH × EH-2) population, as the percentage of a genome that originated from Varuna.

### QTL Analysis

The five data sets (VEH, BC-V, BC-E, MPH-V, and MPH-E) were used separately for QTL mapping. The midparent heterosis data sets were estimated as the deviation of the backcross hybrids (*BC-V* and *BC-E*) from the midparent value (*MPV*), derived as the mean of the corresponding doubled haploid line (*DH*) and the male parent (Varuna or EH-2). The following equations were used for midparent heterosis estimation, following Radoev et al. (2008):

*MPV*_*i*_ = (*DH*_*i*_ + *P1 or P2*)/*2*
*MPH*_*i*_ = *BC*_*i*_ –* MPV*_*i*_

where DH is the VEH doubled haploid line, P1 is Varuna, P2 is EH-2, and BC is a backcross hybrid.

Quantitative trait loci, which contribute to heterosis, are presumably those detected with the *MPH*-data sets.

For each data set, the trait averaged values of three replications in each of the three environments were used for QTL analysis, while the combined analysis was based on BLUPs for each trait across the three environments. QTL mapping was carried out using CIM (composite interval mapping) in Windows QTL Cartographer 2.5 (Wang et al., 2012). For CIM, the standard model (Model 6) was used with forward regression, a window size of 10 cM and five background control markers. The genome was scanned for putative QTL with main effects at a walking speed of 1 cM. The experiment-wise error rate was determined by performing 1,000 permutations to obtain the empirical thresholds (Churchill and Doerge, 1994). We adopted LOD values >3.0 for identifying “significant” QTL. To avoid missing minor QTL, a lower LOD value corresponding to *p* ≤ 0.20 was adopted for the presence of “suggestive” QTL (Shi et al., 2011). For each trait, multiple QTL peaks detected from a single trial within 10 cM of each other were regarded as a single QTL. The QTL were classified as major when their *R*^2^ (phenotypic variation explained) value was more than 10%. The graphical representations of QTL on linkage groups were drawn using MapChart 2.32 software (Voorrips, 2002).

The gene actions in different data sets varied according to the type of the mapping population and the input data. The three types of data sets (DH, BC, and MPH) described above provided different genetic effect information. QTL obtained in the DH population explained the additive effect “a.” The dominance effect (d) was explained by the QTL obtained in two midparent heterosis data sets, MPH-V, and MPH-E, which revealed the heterotic loci. QTL obtained in the backcross populations explained a combination of additive and dominance effects – (a + d)/2 and (a–d)/2 if the donor or the recurrent parent carries a dominant-increasing allele, respectively (Radoev et al., 2008). QTL detected simultaneously in different data sets allowed an assessment of the degree of dominance by calculating d/a ratio. The mode of inheritance of identified QTL was defined as additive (|d/a| <0.2), partially dominant (0.2 ≤ |d/a| <0.8), dominant (0.8 ≤ |d/a| <1.2), and overdominant (|d/a|≥1.2) (Stuber et al., 1987).

To dissect the epistasis (QTL × QTL) involved in the expression of the traits in the VEH, BC-V, BC-E, MPH-V, and MPH-E data sets, software IciMapping 4.0 (Meng et al., 2015) was used. A threshold of LOD ≥ 2.5 (PIN = 0.001) was used for declaring the presence of main-effect QTL (M-QTL), and LOD ≥ 5.0. (PIN = 0.0001) was used for declaring the presence of epistatic QTL (E-QTL) (Shang et al., 2016). In the case of epistasis, the estimated effects in VEH population are the additive × additive genetic interactions, while the effects calculated in the BC-V, BC-E, MPH-V, and MPH-E data sets are complex mixtures of all possible epistatic interactions: additive × additive (aa), additive × dominance (ad), and dominance × dominance (dd) interactions (Radoev et al., 2008).

### Candidate Gene Analysis

Sequences of the SNP markers flanking QTL regions were mapped to the *B. juncea* reference genome (Paritosh et al., 2021; https://www.ncbi.nlm.nih.gov/assembly/GCA_015484525.1), available on NCBI database using BLAST tool (https://blast.ncbi.nlm.nih.gov/Blast.cgi) to project the QTL on the physical map and to find marker positions. Genes that were physically located within the QTL regions were annotated using the TAIR GO term enrichment tool (https://www.arabidopsis.org/tools/go_term_enrichment.jsp), which sends the data to Panther classification system (Ashburner et al., 2000; Thomas et al., 2003; Mi et al., 2010, 2021).

## Results

### Construction of the VEH Linkage Map

A total of 513 polymorphic markers (IP, SSR, and KASP-SNP) were used as anchor markers in the linkage analysis. A further set of 10,007 high throughput SNPs were identified out of the 77,969 markers available on the ABC 90K Illumina chip. Of these, markers with missing data (> 30%), significant segregation distortion, and monomorphic markers were removed. This screen left us with 7,274 robust markers along with the 513 anchor markers that were used for constructing the final map. Out of the 7,787 markers that were selected for genotyping the VEH population, 7,609 markers (7,101 SNPs and 508 anchor markers) showed consistent ordering on the 18 linkage groups (Table 1). The total length of the map was 2,627.9 cM with an average number of 422 markers per linkage group (Supplementary Table 2).

**Table 1:** Characteristic features of the linkage groups in the VEH map, constructed using IP, SSR, KASP-SNP, and Chip-based SNP markers.

**Chromosome**	**Anchor markers**	**Chip SNPs**	**Number of markers**	**Total length (cM)**	**Average spacing (cM)**	**Marker interval**	**Marker density**
A1	33	376	409	110.7	0.3	76	3.7
A2	40	394	434	135.3	0.3	82	3.2
A3	39	388	427	139.1	0.3	93	3.1
A4	27	402	429	104.5	0.2	62	4.1
A5	28	347	375	116.3	0.3	73	3.2
A6	29	181	210	142.7	0.7	72	1.5
A7	21	375	396	138.8	0.4	61	2.9
A8	15	202	217	99.0	0.5	71	2.2
A9	43	568	611	171.6	0.3	104	3.6
A10	12	171	183	122.8	0.7	53	1.5
A Genome	287	3,404	3,691	1280.8	–	747	–
Mean	28.7	340.4	369.1	128.1	0.4	74.7	2.9
B1	24	788	812	112.0	0.1	54	7.3
B2	23	758	781	174.1	0.2	110	4.5
B3	38	816	854	206.7	0.2	133	4.1
B4	28	80	108	162.2	1.5	54	0.7
B5	24	238	262	157.5	0.6	82	1.7
B6	16	176	192	133.3	0.7	62	1.4
B7	36	432	468	154.4	0.3	91	3.0
B8	32	409	441	247.0	0.6	103	1.8
B Genome	221	3,697	3,918	1347.1	–	689	–
Mean	27.6	462.1	489.8	168.4	0.5	86.1	3.1
Total (A+B Genome)	508	7,101	7,609	2627.9	–	1,436	–
Mean	28.2	394.5	422.7	146.0	0.3	79.8	3.0

### Trait Performance of Parents, F_1_, VEH, and Two Backcross Populations

The performance of VEH population, two backcross populations (BC-V and BC-E), parental lines (Varuna and EH-2), and their F_1_ hybrid, along with the levels of midparent heterosis (MPH) observed in the F_1_, BC-V, and BC-E populations for the 14 traits are summarized in Supplementary Table 3. The two parents contrasted for most of the traits, making them ideal candidates for heterosis breeding and related experiments. In the F_1_, significant midparent heterosis was observed for PH, PBR, SBR, SPY, SQD, SQPL, and OIL.

The BC-V population showed significantly high MPH for SBR, SPY, and SQPL, while, in the BC-E population, significantly high MPH was recorded for PBR, SBR, SPY, SQD, and SQPL. Significant genetic variation was observed in most of the traits in the VEH, BC-V, and BC-E populations (Supplementary Table 4). DF, MSL, and PH showed high-genetic variance and heritability in all three trials of the populations. High heritabilities were also observed for OIL, SQD, SQL, and TSW even with low genetic variances in all trials in the VEH as well as in BC-V and BC-E populations. Siliques on the main shoot and siliques on a plant showed high genetic variance but lower heritability in all trials of all populations. The trait PBR showed low genetic variance but high heritability in two of the three trials. PRO showed low genetic variance in all three populations with high heritability in VEH but lower in both BC populations. The traits SBR and SPY showed high-genetic variance in two trials of VEH population but reduced genetic variances in all the trials of the two BC populations. SSQ showed high-genetic variance in VEH population, lower in the BC populations while showing moderate to high heritability in all the populations and trials.

### Correlations Among Traits

Correlation coefficients among the 14 traits were estimated using the phenotypic data of the three environments and BLUPs. Both positive and negative correlations among the traits were observed in all the three populations in the three trials (Supplementary Table 5). In VEH, PH showed significant positive correlations with DF, PBR, SQD, and SQMS. The traits PBR and SBR showed a significant negative correlation with MSL and TSW. Single plant yield showed a significant positive correlation with PBR, SBR, SQMS, SQPL, and TSW. Similar correlations were observed in BC-V and BC-E populations.

#### Correlations Between DH Lines, Backcross Performance, and Midparent Heterosis

In all the traits, a significantly high positive correlation was observed between the performances of VEH and the two BC populations in all three trials (Supplementary Table 6). Phenotypic performance of VEH for most traits was negatively correlated with MPH performances. Performances of BC-V and BC-E populations showed a significantly high positive correlation with the corresponding MPH performances.

#### Relationships Between Traits and Genome Heterozygosity

Relationships between molecular marker heterozygosity and phenotypic performance of the 14 traits in the BC hybrids was also evaluated by regressing the midparent heterosis value and the trait value of each BC hybrid on its percentage of genome heterozygosity. Heterozygosity in the backcross populations showed a mean of 49 and 48% in the BC-V and BC-E populations, respectively. For most traits, genome heterozygosity was found to be positively correlated with the performance of the two BC populations and MPH (Table 2). The correlation coefficients were, however, mostly low in the four data sets (BC-V, MPH-V, BC-E, and MPH-E), indicating that the overall genome heterozygosity alone had little effect on the trait expression.

**Table 2:** Coefficients of correlation between whole-genome heterozygosity and trait performance in the BC and MPH data sets.

**Trait**	**Year**	**BC-V**	**MPH-V**	**BC-E**	**MPH-E**
**Plant architectural traits and days to flowering**					
PH	2014-15	0.276^**^	0.278^**^	−0.049	0.186^*^
	2015-16	0.285^**^	0.332^**^	−0.002	0.171^*^
	2016-17	0.258^**^	0.192^*^	−0.083	0.163
DF	2014-15	0.118	0.105	−0.109	−0.013
	2015-16	0.173^*^	0.204^**^	0.089	−0.091
	2016-17	0.071	0.082	−0.024	0.02
MSL	2014-15	−0.042	0.03	−0.005	−0.114
	2015-16	−0.033	0.041	−0.037	−0.131
	2016-17	−0.1	−0.041	0.002	−0.057
PBR	2014-15	0.171^*^	0.107	−0.009	0.07
	2015-16	0.101	0.031	0.084	0.09
	2016-17	0.186^*^	0.199^*^	0.057	0.167
SBR	2014-15	0.300^**^	0.310^**^	0.087	0.083
	2015-16	0.116	0.099	0.069	0.063
	2016-17	0.081	0.026	0.206^*^	0.222^**^
**Silique related traits**					
SPY	2014-15	0.365^**^	0.377^**^	0.241^**^	0.186^*^
	2015-16	0.082	0.095	0.104	0.07
	2016-17	0.141	0.153	0.102	0.166^*^
SQD	2014-15	0.164^*^	0.062	−0.062	0.170^*^
	2015-16	0.217^**^	0.02	0.002	0.268^**^
	2016-17	0.207^*^	0.15	0.076	0.171^*^
SQL	2014-15	−0.297^**^	0.055	0.343^**^	0.077
	2015-16	−0.377^**^	−0.051	0.379^**^	0.083
	2016-17	−0.283^**^	0.004	0.230^**^	−0.005
SQMS	2014-15	0.159^*^	0.101	−0.08	0.068
	2015-16	0.189^*^	0.09	−0.044	0.078
	2016-17	0.174^*^	0.189^*^	0.122	0.145
SQP	2014-15	0.397^**^	0.323^**^	0.126	0.133
	2015-16	0.261^**^	0.143	0.089	0.135
	2016-17	0.222^**^	0.170^*^	0.046	0.15
SSQ	2014-15	0.276^**^	0.145	−0.034	0.016
	2015-16	0.231^**^	0.107	−0.005	0.072
	2016-17	0.212^*^	−0.024	−0.083	0.133
**Seed related traits**					
OIL	2014-15	0.270^**^	0.300^**^	0.084	0.164^*^
	2015-16	0.069	0.141	0.092	0.114
	2016-17	0.192^*^	0.228^**^	0.065	0.13
PRO	2014-15	−0.115	−0.201^**^	−0.168^*^	−0.126
	2015-16	−0.156^*^	−0.276^**^	−0.237^**^	−0.174^*^
	2016-17	−0.082	−0.239^**^	−0.215^*^	−0.113
TSW	2014-15	−0.349^**^	−0.108	0.225^**^	−0.123
	2015-16	−0.418^**^	−0.248^**^	0.223^**^	−0.127
	2016-17	−0.350^**^	−0.142	0.187^*^	−0.031

* *Significant at 5%*,

** *Significant at 1%*.

### Main-Effect QTL

A total of 695 main-effect QTL were detected for the 14 traits in the three trials using the five data sets (Supplementary Table 7). Of these, 176 QTL were detected in VEH, 188 in BC-V, 164 in BC-E, 92 in MPH-V, and 75 in the MPH-E data set. Approximately 75% of these 695 QTL were significant and were identified with LOD values >3, while the remaining proportion consisted of suggestive QTL with LOD values ranging between 2.5 and 3.

A highest number of QTL were detected on the linkage groups B3 (86), A7 (84), and A3 (77) while the lowest number of QTL was detected on B6 (5). The total number of major QTL explaining a phenotypic variance of more than 10% was 175, with the highest number of these mapping to A7 (Supplementary Table 7). A summary of heterotic QTL and their effects is shown in Table 3. Overdominance was observed to be the predominant type of effect in the heterotic QTL.

**Table 3:** Effects of heterosis QTL identified for 14 yield traits in two backcross populations in the three trials.

		**BC-V**				**BC-E**			
	**Trait**	**A**	**PD**	**D**	**OD**	**A**	**PD**	**D**	**OD**
Plant architectural traits and days to flowering	PH	9	2	3	13	6	2	1	10
	DF	14	2	4	11	9	1	2	5
	MSL	8	1	1	3	8	0	2	9
	PBR	6	0	0	9	5	0	0	5
	SBR	8	0	0	9	6	0	0	5
Silique related traits	SPY	3	0	0	7	6	0	0	6
	SQD	8	0	1	8	8	0	1	11
	SQL	14	1	3	9	6	1	4	5
	SQMS	7	0	0	4	2	0	0	5
	SQPL	6	0	1	7	3	0	1	7
	SSQ	7	0	2	3	5	2	1	2
Seed related traits	OIL	8	3	1	7	7	0	2	9
	PRO	8	0	1	5	11	1	0	4
	TSW	6	0	0	13	13	1	0	4
	Total	112	9	17	108	95	8	14	87

*A, additive effect QTL; PD, partial dominance effect QTL; D, dominance effect QTL; and OD, over dominance effect QTL*.

The combined analysis using BLUPs identified a total of 281 QTL from the five data sets, of which approximately 68% were identified as significant with LOD values >3. The largest number of QTL was detected in VEH population with a total of 72 QTL. Similar to the number of QTL identified with MPH-E data set in the single environments, MPH-E detected the least number of 31 QTL in the combined analysis also (Supplementary Table 8).

### Plant Architectural Traits and Days to Flowering

#### Plant Height

Twenty-two QTL with additive effects were detected in the VEH population in the three trials. The QTL on LG A07 were consistently identified in all three trials and showed a large positive additive effect with substantial proportions of phenotypic variance explained (PVE). For all other QTL detected for PH, the additive effects were mostly negative, indicating that the alleles for taller plants were contributed by the parent EH-2 (Supplementary Table 7).

A total of 21 and 17 QTL were identified in the BC-V and BC-E populations of backcross hybrids, respectively, in the three trials. All of the six common QTL detected in the BC-V, BC-E, and VEH populations showed considerably larger effects in the backcross populations compared with the additive effects observed in VEH at these loci, indicating non-additive gene action. The two MPH data sets detected 14 QTL, of which three QTL in MPH-V and two in MPH-E were also found in other data sets. The effects in the two MPH data sets were mostly positive, suggesting that these QTL in heterozygous conditions would increase PH with respect to the midparent value, consistent with the positive heterosis observed for this trait. For QTL detected simultaneously in the different data sets, degree of dominance could be calculated, which revealed that the trait is controlled by a combination of mostly overdominant loci with a few partially dominant loci.

#### Days to Flowering

Seventeen QTL were mapped in the VEH population in the three trials, 14 of which showed negative additive effects, indicating that Varuna contributed the alleles for an earlier flowering time. Consistent with the highly significant correlations between DF and PH, major QTL on the LG A07 were identified as common QTL for both the traits, with Varuna contributing the positive alleles for taller plants and delayed flowering. The two backcross populations identified a total of 35 QTL in the three trials. Fifteen QTL in BC-V and 11 QTL in BC-E showed negative effects, indicating that the alleles for delayed flowering in the hybrid combination were provided by EH-2. The QTL analysis for the two MPH data sets identified 12 QTL with PVEs ranging from 5 to 12.5%. Two of the major QTL for DF detected in MPH-V showed dominance effects with opposite signs. Thus, the counterbalancing dominance effects along with the additive effects explain the absence of significant heterosis in DF.

#### Main Shoot Length

Twelve QTL were detected for MSL in the VEH population, which explained PVEs ranging from 4.3 to 15.3% in the three trials. In seven cases, the effects were positive, implying that Varuna contributed the positive alleles for MSL at these loci. Twenty-four QTL were mapped with the two backcross data sets, and the QTL on LGs A04, A07, and B03 were also identified in the doubled haploid (VEH) population. Ten QTL were detected with the two MPH data sets with an equal number of loci showing positive and negative dominance effects, thus, resulting in the low heterosis exhibited by the trait.

#### Number of Primary Branches (PBR)

A total of 38 QTL were identified, including 16 major QTL in the five data sets. Fifteen QTL were detected in the VEH population for which both the parents, Varuna, and EH-2 contributed positive alleles. Of the 19 QTL mapped in the two backcross populations, 14 showed negative effects, indicating that the increasing alleles were provided by EH-2. Fourteen QTL were identified using the MPH-V and MPH-E data sets, and the effects were mostly positive, consistent with the significant heterosis observed for PBR. The QTL on LG A07 were consistently detected as major QTL with high PVEs in BC-V, BC-E, VEH, and MPH-E data sets, wherein Varuna increased the number of primary branches in both homozygous and heterozygous conditions at this locus.

#### Number of Secondary Branches (SBR)

An equal number of seven loci each, with positive and negative effects among the total of 14 QTL, were detected for SBR, indicating the dispersal of increasing alleles in both Varuna and EH-2. Of the 17 QTL mapped in the BC-V and BC-E populations, 13 showed negative effects, indicating that EH-2 contributed the trait-increasing alleles for number of secondary branches at these loci. Eleven QTL were identified in the two MPH data sets, seven of which showed overdominance as they were not identified in the backcross and DH data sets.

### Silique-Related Traits

#### Single Plant Yield

Ten QTL were mapped with the VEH data set, with both Varuna and EH-2 contributing the positive alleles at an equal number of five loci each (Supplementary Table 7). Three of these were identified as major QTL, explaining more than 10% of the PVE, and were located on the LGs A04, B02, and B04. The two backcross data sets detected 16 QTL, of which five were detected as major QTL. At four of these five major QTL identified in the BC-V and BC-E populations, Varuna provided the increasing dominant allele. Fifteen dominant QTL were detected with the MPH-V and MPH-E data sets, and 13 of these showed positive dominance effects, implying that the heterozygotes exhibited higher single plant yields congruent with the highly significant amount of heterosis observed for SPY. The effects of these dominant loci were also appreciably greater than the effects observed in the DH (VEH) population, indicating their overdominant nature.

#### Silique Density

Twelve QTL affecting SQD were detected in the VEH population in the three trials, with the higher parent (EH-2) donating the increasing allele at eight of these loci. The two backcross populations detected 29 QTL in the three trials, which were distributed as 13 loci in BC-V and 15 loci in BC-E, along with a common locus for the trait. The effects were mostly negative in BC-V and positive in BC-E, indicating that EH-2 and Varuna provided the trait-enhancing alleles as donor parents in BC-V and BC-E, respectively. Fourteen QTL were mapped with the MPH-V and MPH-E data sets, and nine of these showed positive dominance effects, indicating that these loci would increase SQD in heterozygous state. This observation is in consonance with the positive heterosis shown by the trait. Furthermore, seven of the 14 QTL detected with the two MPH data sets showed overdominance as no corresponding QTL were identified with additive effects in other data sets.

#### Silique Length

Fifteen QTL with additive effects were identified in the VEH population in the three trials. Except the QTL on the LG A01, which received the trait-increasing allele from EH-2, all the remaining 14 QTL inherited the trait-increasing allele for SQL from the higher parent, Varuna. Most of the 28 QTL identified in the two backcross populations also showed positive effects, indicating the contribution of the increasing-dominant allele by Varuna as the recurrent parent in BC-V and as the donor parent in the BC-E hybrids. Nine QTL were detected in the MPH-V and MPH-E data sets, of which three over-dominant QTL showed negative dominance effects. The low heterosis observed for SQL was thus attributed to QTL, with mostly additive effects and a few other over-dominant loci with opposite signs.

#### Siliques on the Main Shoot

Six QTL were detected in the VEH population in the three trials, of which four QTL showed negative effects, indicating the contribution of positive alleles by EH-2 toward increasing siliques on the main shoot. Fourteen QTL were detected in the two backcross populations, of which 10 QTL showed negative effects, implying the role of EH-2 in donating the trait-enhancing alleles as the donor parent in BC-V and as the recurrent parent in BC-E population. Eleven QTL were identified in the MPH-V and MPH-E data sets, all of which were considered as overdominant QTL. Two major QTL with large effects were detected in the LGs A05 and B02, with opposite signs. Based on the QTL effects, SQMS appeared to be influenced largely by additive effects in combination with a few overdominant loci, which showed counterbalancing effects.

#### Siliques on a Plant

All the seven QTL identified in the VEH population invariably showed negative additive effects, indicating that the higher parent EH-2 contributed the alleles at these loci for an increase in the number of siliques on a plant. In the two backcross populations, 10 QTL were detected in the BC-V population compared with only four in the BC-E population. All the QTL in the BC-V population along with the two QTL identified in the BC-E population exhibited negative effects, implying that EH-2 provided the increasing dominant alleles at these loci as the donor parent in BC-V and as the recurrent parent in BC-E. Most importantly, a total of 13 QTL identified with the two MPH data sets showed positive dominant effects along with QTL on LG B02, which showed a negative dominance effect. The QTL identified in the MPH-E data set and mapping to LG A09 exhibited the largest effect, which was about six times the effect observed for the smallest effect QTL in the VEH population. The significantly high heterosis observed for SQPL can be explained with a large number of loci exhibiting overdominance.

#### Seeds in a Silique

Seven QTL with PVEs ranging from 6.3 to 22.2% were identified in the VEH population in the three trials. Six of these, including the major QTL, showed negative effects, indicating that these QTL inherited the alleles for the increased number of seeds in a silique from the higher parent, EH-2. The two backcross populations detected a total of 18 QTL, including six major QTL with PVEs >10%. Similar to the results obtained with the VEH population, a majority of 15 QTL in the BC-V and BC-E displayed negative effects, implying that EH-2 contributed to the increasing dominant alleles in both populations. The QTL mapping with the two MPH data sets resulted in the detection of six minor QTL, all of which showed negative dominance effects. The trait of SSQ is most probably controlled by loci with additive effects as the number of QTL with dominance effects was less as compared with the loci with additive effects, an observation consistent with the insignificant heterosis observed for SSQ.

### Seed-Related Traits

#### Oil Content

Eleven QTL with significant additive effects were mapped in the VEH population in the three trials, with an approximately equal number of six and five positive alleles for increased oil content being contributed by Varuna and EH-2, respectively (Supplementary Table 7). The QTL on the linkage groups on A08 and B07 were consistently detected in all the three trials. The genomic regions harboring these QTL have previously been shown to contain the erucic acid genes (Paritosh et al., 2014; Rout et al., 2018). All the five QTL in these regions showed positive effects, indicating that Varuna contributed to the increasing alleles. For the remaining six QTL, EH-2 donated the positive alleles. A total of 24 QTL were detected in the two backcross populations in the three trials, two-thirds of which showed negative effects, indicating the contribution of EH-2 in increasing oil content in the backcross hybrids. The QTL analysis with the two MPH data sets revealed the existence of 17 QTL, showing dominance effects. Of these 17 loci, a majority of 14 QTL showed positive dominance effects, indicating that heterozygosity at these QTL would increase oil content, which was congruent with the significant levels of heterosis for this trait. For the QTL on the LGs A08 and B07, both the additive and dominance effects were positive, meaning that, at these loci, the Varuna allele for increasing oil content was dominant.

#### Protein Content

Of the 14 QTL identified for PRO in the VEH population in the three trials, 10 showed negative effects, indicating that EH-2 contributed alleles for higher protein content in the doubled haploid lines. Twenty-three QTL were identified in the two backcross populations in the three trials. While the effects were mostly positive in BC-V, the effects in BC-E were negative, indicating that the recurrent genotype had a major influence on the protein content of the backcross hybrids. A total of six QTL were mapped using the two MPH data sets, four of which showed negative dominance effects in agreement with the negative but insignificant heterosis observed for the trait in two of the three trials. The QTL on LG A03 showed both the positive additive and dominance effects, indicating that the Varuna allele-increasing protein content was dominant. The mode of genetic action thus appears to be mostly due to additive loci along with a few dominant loci with opposite signs.

#### Thousand Seed Weight

Fourteen QTL were mapped in the VEH population, a majority of which showed positive effects, indicating that the parent Varuna contributed the favorable alleles. Twenty-seven QTL were detected in the BC-V and BC-E hybrids and explained PVEs ranging from 4.8 to 19.5%. The genetic effects observed in the BC-V population were invariably positive, while, in the BC-E population, 13 of the 17 QTL showed positive effects. The prevalence of the positive effects in the two backcross populations can be ascribed to the trait-increasing alleles provided by the higher parent, Varuna. A comparatively greater number of 10 QTL were identified using the MPH-V data set compared with only two with the MPH-E data set. In MPH-V, all the QTL, excluding one, displayed negative dominance effects, indicating that these QTL in the heterozygous state would reduce seed weight. Conversely, both the QTL detected with MPH-E data set exhibited positive dominance effects, connoting a positive influence of heterozygosity on seed weight at these loci. However, the negative dominance effects can be presumed to result in the observed negative heterosis for seed weight. The mode of action governing TSW in *B. juncea* thus appears to be a combination of additive effects and QTL in the dominance-overdominance range.

### Environmentally Stable QTLs

Quantitative trait loci, which were detected within two or all of the three environments for the five data sets, and also in the combined analysis based on BLUPs, were considered as “environmentally stable QTL.” Based on this criterion, a total of 88 environmentally stable QTL were identified for all the 14 traits in the five data sets (Supplementary Table 8). The largest number of 11 environmentally stable QTL was detected for PH, while only two QTL for SQMS were identified concurrently in the combined analysis and in at least two of the three environments. Consistent with the QTL analysis in the three individual environments, the genetic intervals on A07 were identified as the environmentally stable QTL for all the traits, excluding SQMS and PRO.

### Epistatic QTL

A total of 637 epistatic QTL (E-QTL) were detected in the five data sets for the 14 traits (Supplementary Table 9). Out of these, 3 interactions were found to be between two main-effect QTL (Type I interaction), 102 interactions between main-effect QTL and QTL without any significant main effect (Type II interaction), and 532 interactions were between two QTL without any significant main effect (Type III interaction) (Table 4).

**Table 4:** Type of epistatic interactions detected in the five data sets in 14 traits.

**Trait**	**Population/Data set**	**Type 1**	**Type 2**	**Type 3**	**Total E-QTL**
**Plant architectural traits and days to flowering**				
PH	BC-V	0	5	18	23
	BC-E	0	5	19	24
	VEH	1	8	23	32
	MPH-V	0	0	5	5
	MPH-E	0	1	2	3
DF	BC-V	0	2	6	8
	BC-E	0	3	12	15
	VEH	0	6	24	30
	MPH-V	0	1	7	8
	MPH-E	0	0	6	6
MSL	BC-V	0	0	7	7
	BC-E	0	1	4	5
	VEH	0	2	22	24
	MPH-V	0	0	2	2
	MPH-E	0	0	0	0
PBR	BC-V	1	5	9	15
	BC-E	0	3	4	7
	VEH	0	1	9	10
	MPH-V	0	0	5	5
	MPH-E	0	0	0	0
SBR	BC-V	0	3	14	17
	BC-E	0	0	6	6
	VEH	0	5	16	21
	MPH-V	0	3	11	14
	MPH-E	0	0	1	1
**Silique related traits**				
SPY	BC-V	0	0	8	8
	BC-E	0	0	5	5
	VEH	0	2	4	6
	MPH-V	0	0	2	2
	MPH-E	0	0	2	2
SQD	BC-V	0	2	5	7
	BC-E	0	1	9	10
	VEH	0	2	14	16
	MPH-V	0	0	1	1
	MPH-E	0	0	2	2
SQL	BC-V	0	3	4	7
	BC-E	0	2	6	8
	VEH	0	2	10	12
	MPH-V	0	0	3	3
	MPH-E	0	0	1	1
SQMS	BC-V	0	1	17	18
	BC-E	0	0	3	3
	VEH	0	1	23	24
	MPH-V	0	1	8	9
	MPH-E	0	0	1	1
SQP	BC-V	0	3	4	7
	BC-E	0	0	10	10
	VEH	0	0	4	4
	MPH-V	0	0	3	3
	MPH-E	0	0	2	2
SSQ	BC-V	0	0	12	12
	BC-E	0	2	7	9
	VEH	0	8	33	41
	MPH-V	0	0	4	4
	MPH-E	0	0	5	5
**Seed related traits**				
OIL	BC-V	0	1	5	6
	BC-E	0	1	9	10
	VEH	0	3	12	15
	MPH-V	0	0	5	5
	MPH-E	0	2	1	3
PRO	BC-V	0	2	4	6
	BC-E	0	1	7	8
	VEH	0	2	9	11
	MPH-V	0	0	4	4
	MPH-E	0	0	4	4
TSW	BC-V	1	1	10	12
	BC-E	0	0	2	2
	VEH	0	5	11	16
	MPH-V	0	0	4	4
	MPH-E	0	0	1	1
Total		3	102	532	637

In the VEH population, a total of 262 epistatic interactions involving 505 loci were identified with PVEs in the range of 0.4–8.7%. The average PVE of the epistatic interactions was 2.8%. Of the 14 traits analyzed for epistasis in the VEH population, SSQ was characterized by the highest number of 41 interactions, while SQPL detected only 4 interactions. SSQ also showed the highest number of 8 Type II and 33 Type III interactions. A total of 49 main-effect QTL were observed to contribute to the Type I and Type II interactions. Only a single Type I interaction was identified in the VEH population, which was identified for PH. All of the digenic interactions observed for SPY exhibited negative effects, denoting recombinant allele combinations increased SPY.

Totally, 275 interactions were identified in the BC-V and BC-E populations, with an average PVE of 2.4% in each of these two populations. The PVEs by the interacting loci were in the range of 0.07 to 5.7% in BC-V and 0.6 to 4.4% in the BC-E population. In the BC-V population, a total of 295 loci were involved in 2, 14, and 123 interactions belonging to Types I, II, and III, respectively. In comparison, 236 loci were involved in a total of 122 epistatic interactions of Types II and III in the BC-E population. The interacting E-QTL involved 32 and 19 QTL with main effects in the BC-V and BC-E populations, respectively. Notably, PH showed the highest number of interactions in both the backcross populations, majority of which were Type III, occurring between background loci with no significant main effects. The traits of SPY and SQMS were characterized by negative epistatic interactions, indicating that the recombination among the parental alleles would increase SPY and SQMS.

The two MPH data sets, MPH-V, and MPH-E individually detected 69 and 31 digenic interactions, respectively. The interactions identified using the MPH-V data set exhibited PVEs in the range of 0.06 to 5.6%, with an average of 2.9%, while the loci involved in interactions identified using the MPH-E data set showed PVEs, ranging from 0.09 to 5.7%, with an average of 2.9%, similar to that observed with the MPH-V data set. The traits of SBR in MPH-V and DF in the MPH-E data set displayed the largest number of 14 and 6 interactions, respectively. The traits, SPY and SQPL in the MPH-V, and SBR and SQMS in MPH-E included interactions with negative effects only, meaning the recombinant allele combinations favored increases in the phenotypes of these traits. Ninety-three percent of the interactions, which were detected using the two MPH data sets, were of Type III, occurring between background loci without significant main effects, while the remaining 7% were of Type II and involved a combination of QTL with main effect and a modifying background locus.

### Active Regions

Quantitative trait loci clusters or hotspots were also identified for the 14 yield and related traits, using the five data sets of all the three trials (Supplementary Figure 1). QTL hotspots have been previously defined as active regions (Basunanda et al., 2010). A total of 59 such active regions were detected in this study, with a maximum of five such regions, each mapping to LGs A09 and B08. The most notable active region was found on the LG A7, which consisted of 79 QTL in a genetic interval of 0.0 cM-72.8 cM. It harbored QTL for 12 of the 14 traits, excluding SQMS and SQPL. Another dense active region was detected on LG A03, consisting of 75 QTL, located in the genetic interval from 20.3 cM to 138.8 cM and had QTL from all the 14 traits. An active region, which included 65 QTL from 13 traits, was also identified on LG B03, the only exception being QTL for SQL. It appears that these regions harbor “reference” or “housekeeping” genes that are expressed during the entire life cycle of the plant by influencing a large number of traits related to plant architecture, flowering, etc. (Basunanda et al., 2010).

Quantitative trait loci analysis performed with BLUPs also showed the presence of an active region on A7 and resulted in a more precise interval of 0.0–11.2 cM with QTL for 8 of the 14 traits. This region, consisting of QTL for days to flowering, plant architectural traits, silique- and seed-related traits was therefore, analyzed *in silico* to identify putative candidate genes. Based on the annotation of *Arabidopsis* genome, 288 genes were identified, which were subsequently analyzed using TAIR GO term enrichment tool. The analysis revealed a total of 67 significant genes (*p* < 0.05) under the GO Biological processes category, which included MYB122 (AT1G74080), bHLH160 (AT1G71200), NAC031 (AT1G76420), MYB54 (AT1G73410), and other transcription factors (Riechmann et al., 2000; Stracke et al., 2001; Vroemen et al., 2003), gene-encoding single-stranded DNA-binding protein WHY2 (AT1G71260), which is a mitochondrial member of the WHIRLY family of plant-specific proteins (Desveaux et al., 2005), WAT1 (AT1G75500), which is required for secondary wall formation in wood fibers (Ranocha et al., 2013), PIN1(AT1G73590), and PIN3 (AT1G70940), both of which act as components of the auxin efflux carrier (Zhou and Luo, 2018) (Supplementary Table 10).

## Discussion

### Advantages of the Experimental Design for Genetic Analysis of Heterosis

This study provides the first report on the use of different segregating populations from the same parental combination to study the genetics of heterosis for yield in *B. juncea*. The two inbred parents, Varuna and EH-2, have been shown to be good heterotic combiners, as the F_1_ hybrid from this combination (hybrid DMH-11) shows about 28–36% yield heterosis in plot-level yield trials (personal communication). The unique experimental strategy of using the DH and the two BCF_1_ (BC-V and BC-E) populations was specifically designed to accurately resolve different types of gene action along with a comprehensive mapping of loci contributing to yield-influencing traits in *B. juncea*. The two backcross populations can be repeatedly developed from the DH lines, and the genotypes of the backcross hybrids can be unambiguously deduced from the marker information of the parental doubled haploid lines, thus obviating the need for additional genotyping and mapping procedures. A similar design was earlier adopted by Radoev et al. (2008) and Basunanda et al. (2010) for studying heterosis for seedling biomass and yield traits.

Zhihong et al. (2012) have also highlighted the advantages of this design, also known as the double backcross design and is used for constituting an immortal mapping population, allowing for across-environment replications, estimation of dominance effects, epistatic effects, and QTL-environment interactions, and thus remedies the drawbacks of a single backcross population. Recent studies on QTL mapping in rice, maize, cotton, and *B. napus* have shown that dominance and epistasis, especially the additive × additive interaction, play a key role in contributing to heterosis (Stuber et al., 1992; Yu et al., 1997; Tang et al., 2010). Using a conventional BC population, the additive × additive interactions cannot be detected separately because epistasis is identified as mixtures of additive and dominance effects. The double backcross population design obviates this problem by accurately and efficiently estimating additive × additive interaction effects.

The results obtained in this study revealed that the design in the present study was more efficient to map common stable QTL and several heterozygous loci across multiple populations than that could be possibly detected from a single-base population. However, the backcross populations could account for only 50% of the possible heterosis. Use of the two BC populations was also made possible to clearly detect dominant QTL from both the parents, which would otherwise have been difficult if we had used independent testers (Mei et al., 2003).

### A High-Density Linkage Map in *B. juncea*

Several linkage maps have been constructed in *B. juncea* using mapping populations with various genetic structures based on a wide array of molecular markers, and a number of QTL for yield and quality traits have also been identified from these linkage maps (Ramchiary et al., 2007; Pradhan and Pental, 2011; Yadava et al., 2012; Yang et al., 2016; Dhaka et al., 2017; Rout et al., 2018; Paritosh et al., 2021). However, heterosis for yield and yield component traits has not been analyzed using any of these maps reported so far. The VEH linkage map includes a large number of SNPs, which are the most frequent type of genetic polymorphisms, providing a high density of markers near a locus of interest (Picoult-Newberg et al., 1999). The high-density map used in this study was based on SNP markers identified using ABC 90k Illumina SNP array (Mason et al., 2017). The well-characterized sequence-based markers used in the array, along with the availability of *B. juncea* genome sequence, ensure the efficient dissection and subsequent use of complex traits in Brassica breeding programs.

The Illumina 60K Brassica SNP Bead Chip array (Clarke et al., 2016) has previously been used in QTL analysis in *Brassica napus* to study flowering time, seeds per silique, silique length, seed weight, and other traits (Yang et al., 2017; Wu et al., 2018; Wang et al., 2019; Song et al., 2021) but has not yet been utilized in genetic mapping of heterotic loci. Because *B. juncea* and *B. napus* share A genome, therefore using common markers, an attempt was made to align QTL for yield-related traits detected in the present study with those detected in previous studies undertaken in *B. napus*. However, only one QTL detected for seeds per silique on linkage group A07 with PVE >10% (Supplementary Table 7) could be aligned with QTL (qSSA07.4) identified by Yang et al. (2017). Furthermore, the IP and SSR markers used in the present study have previously been used to construct a high-density VH map and different integrated maps using multiple populations for studying various seed and oil traits. Based on common markers, three QTL detected for thousand seed weight—one each on A3 (PVE >10%, 102.8–113 cM), A4 (60.9–75.5 cM), and A7 (48.2–64.2 cM)—were aligned with the QTL detected previously by Dhaka et al. (2017), and three oil-content QTL—one each on A7 (10.9–24.1 cM), A8 (12.6–42.2 cM), B7 (77–93.6 cM)—were aligned with QTL detected previously by Rout et al. (2018). The high-density map of *B. juncea* developed in this study will, therefore, facilitate QTL congruency studies by evaluating QTL detected in different mapping populations.

### Mapping QTL With Additive and Dominance Effects

In this study, the performance of BCF_1_ hybrids of the BC-V and BC-E populations was compared with the performance of the parental DH lines (VEH) based on both the phenotypic correlations (Supplementary Table 5) and QTL analyses (Supplementary Table 7). A total of distinct 336 additive QTL were identified for 14 traits in the DH and two BC populations in the three experiments. Of these, 100 were identified only in the DH population, while 131 and 105 QTL were unique to the BC-V and BC-E populations, respectively. Thus, Varuna was the superior tester than EH-2, which probably carried dominant alleles at several loci, and thus, reduced the ability to detect QTL in the BC-E data set. Twenty-six QTL showed strong additive effects and were consistently identified among the three (VEH, BC-V, and BC-E) data sets, while only seven QTL were observed to be common between the BC-V and BC-E populations, which can be primarily attributed to the mode of prevailing gene action (Supplementary Table 7). For these common QTL detected in the three data sets, the direction of contribution was identical. These findings are consistent with the assumption that, in case of the additive model, the heterozygote is exactly halfway between the two homozygotes, and hence, the effect of allele substitution can be revealed in more than one TC population (Melchinger et al., 1998; Frascaroli et al., 2007, 2009).

The BLUP method of analysis (Henderson, 1974) considers fixed environmental and random genetic effects at the same time, and therefore, improves the accuracy of the BLUP values for phenotypic data obtained in different years, different locations, and of progenies of different generations (Piepho et al., 2008; Wang et al., 2016). Using combined QTL analysis based on the BLUP values across three environments, we were able to map environmentally stable QTL. Although a comparatively reduced number of QTL were detected in the combined analyses, the QTL identified using BLUPs corroborated the analyses based on single environments, and a majority of the QTL (78%) were common with the QTL obtained in the analyses of at least one environment (Supplementary Tables 7, 8). In the combined analysis, 51 significant QTL were detected using the BC-V data set compared with 41 significant QTL detected with BC-E data set, indicating the superiority of Varuna over EH-2 as a tester in the current study.

A number of additive QTL were detected either in the BC populations or in VEH population. For QTL detected in the DH population but not in the BC-V, BC-E or the two MPH data sets, the dominant allele carried by the recurrent parent can be assumed to be fully dominant over the donor parent, and *vice versa*. The effects at these loci are based on the difference between the additive and dominance effects, which cancel each other in case of full dominance or they are too low to be detected in case of partial dominance. Conversely, there were QTL observed only in the BC-V and BC-E hybrids (and not in the VEH and the two MPH data sets) (Supplementary Table 7). The genetic effects for these loci represent the sum of the additive and dominance effects, which are probably, too low to be detected separately in the other data sets (Radoev et al., 2008).

Heterosis can be estimated over better parent also when it is called better parent heterosis. In the current study, we have, however, identified QTL using the two MPH data sets (MPH-V and MPH-E) only, as midparent heterosis is one of the most adopted ways of reporting heterosis. For a deeper understanding of the genetic causes of heterosis, comparison of the hybrid with the average performance of both parental lines (and not with only the better parent) remains the most acceptable strategy because the F_1_ hybrid inherits half its nuclear genome from each parent (Melchinger et al., 2007).

A comparatively smaller number of QTL for all the traits were detected in the MPH-V and MPH-E data sets with a total of 92 and 74 QTL, respectively. One of the reasons for this may be the presence of QTL with an intermediate mode of inheritance, which, lacking dominance, could not be detected in MPH data. Secondly, QTL with additive effects larger than dominance effects, that is, QTL with partial or incomplete dominance, were less likely to be identified in MPH data than in the VEH and BC-V or BC-E data (Radoev et al., 2008; Shang et al., 2016). A total of 79 QTL identified in the two MPH data sets were congruent with loci identified with the other data sets, which allowed the assessment of the degree of dominance (Table 3). Of the 134 dominance effects estimated in the BC-V population in the three trials, 9 showed partial dominance, 17 showed dominance, and the remaining 108 exhibited overdominance. In BC-E, out of 109 dominance effects that were estimated, 8 showed partial dominance, 14 showed dominance, while the remaining 87 showed overdominance. A number of QTL identified exclusively with MPH data sets and loci, exhibiting dominance effects higher than the lowest additive effect identified for a trait with VEH population, were suggestive of overdominance. Single-plant yield, which showed the highest level of heterosis, was characterized by a total of six overdominant QTL in the two MPH data sets. In *B. napus*, the largest number of loci exhibiting overdominance was identified for grain yield (Radoev et al., 2008), and seed yield and seed number per plant (Shi et al., 2011) showing the highest level of midparent heterosis of these traits.

In a study on the identification of QTL involved in the control of heterosis in maize, Frascaroli et al. (2007) observed that QTL for traits with low heterosis were prevailingly in the additive to a dominance range, while QTL for highly heterotic traits were characterized by effects in the dominance to overdominance range. However, Shang et al. (2016), while studying the genetic basis of heterosis in Upland cotton using two connected RIL populations and two corresponding BCF_1_ populations, observed that partial dominance and epistasis played a relatively more important role than other genetic effects in heterosis in Upland cotton. Studies on maize (Stuber et al., 1992) and (Frascaroli et al., 2007) have also highlighted the role of overdominance in heterosis. Overdominance has also been identified as the primary genetic basis of heterosis in rice (Li et al., 2001; Luo et al., 2001) and tomato (Krieger et al., 2010). The results in this study show that, although all levels of dominance effects are responsible for heterosis, overdominance is the major contributing factor in the heterosis in *B. juncea* (Table 3). Similar results were reported by Radoev et al. (2008) in *B. napus*, in which the phenotypic variances explained by 14 QTL, showing overdominance, were much larger than that shown by 13 QTL, exhibiting partial- to full-dominance loci for the nine heterotic traits.

Previous studies on *B. napus* (Quijada et al., 2006; Udall et al., 2006; Chen et al., 2007; Basunanda et al., 2010) have also identified heterosis-related QTL clusters, influencing yield-related traits in different DH and/or testcross populations. The active region mapped in the marker interval Bn-A07-p21095697-Bn-A07-p18983802 in linkage group A07 was consistently detected as major QTL in individual trials as well as using the BLUPs, indicating the authentic nature of this QTL and, therefore, should be a focus for further in-depth studies on yield-related traits in *B. juncea* and related species.

### Whole-Genome Heterozygosity and Trait Performance

It was observed in the present study that not all the traits showed higher phenotypes in the backcross lines (BC-V and BC-E heterozygotes) than in their respective DH (VEH homozygotes). QTL detected in both the MPH data sets were a mixture of overdominant and underdominant loci, connoting heterozygosity was not always necessarily advantageous for the expression of the trait. Although significant correlations of whole-genome heterozygosity were observed with some of the traits in the backcross populations and with their respective MPH data sets, the magnitudes of correlations were low (Table 2). It, therefore, appears that heterosis in *B. juncea* arises mainly from heterozygosity at specific loci rather than whole-genome heterozygosity. Similar results were found previously in rice (Yu et al., 1997; Hua et al., 2002; Mei et al., 2005; Luo et al., 2009), upland cotton (Shang et al., 2016), and rapeseed (Radoev et al., 2008). Evidently, the low correlations could also exist due to sparsity of markers or a 50% reduced representation of heterozygous loci in backcross progenies, but, with a higher density of markers and increased genome coverage in the VEH map, the results in this study could be more accurate than other studies, which lacked these features of genetic maps. Large-scale genome-wide association study (GWAS) by Huang et al. (2015) investigated population-scale genomic landscapes from a representative number of hybrid varieties of rice (a predominantly self-pollinated crop) along with parental lines and showed that overall heterozygosity made only small contribution to heterosis. It was concluded that the accumulation of several rare superior alleles with positive dominance contributes to the expression of heterosis in the rice hybrids.

### Mapping QTL With Epistatic Effects

A large number of epistatic interactions were detected in the present study, indicating that epistasis plays an important role in the expression of heterosis in *B. juncea*. The results follow the findings in previous studies on heterosis in rapeseed (Radoev et al., 2008) and Upland cotton (Shang et al., 2016) in which a large number of digenic epistatic interactions were also identified. In studies conducted on maize by Stuber et al. (1992), Lu et al. (2003), and Mihaljevic et al. (2005), and on rice by Xiao et al. (1995), no significant epistasis was detected. In Arabidopsis, Kusterer et al. (2007) found a significant role of epistasis in the expression of heterosis for five biomass-related traits in a triple-testcross design with a recombinant inbred line population. Melchinger et al. (2007) also confirmed the role of epistasis in the expression of heterosis in Arabidopsis, using NIL libraries.

In this study, out of 637 epistatic interactions detected, the vast majority of 532 (83.5%) interactions belonged to the type III class, while 102 (16%) interactions were identified as type II. Only three (0.5%) type I interactions were detected collectively for all the 14 traits using the five data sets (Table 4). In several studies on understanding the genetic basis of heterosis in autogamous species like Arabidopsis (Kusterer et al., 2007; Melchinger et al., 2007), rice (Yu et al., 1997; Li et al., 2001, 2008; Luo et al., 2001; Mei et al., 2005) and barley (Xu and Jia, 2007), epistasis between background loci has been shown to play a major role in comparison to the contribution of loci with main effects, whereas loci with main effects have been ascribed a principal role in determining heterosis in autogamous species such as maize (Stuber et al., 1992; Lu et al., 2003; Frascaroli et al., 2007; Tang et al., 2010; Yi et al., 2019; Xiao et al., 2021). The difference in the genetic basis of heterosis, therefore, appears to be associated with the mode of pollination – open or self-pollination. Radoev et al. (2008), Basunanda et al. (2010), Shi et al. (2011) and Li et al. (2012) have also reported the predominance of epistatic interactions in influencing heterosis in *B. napus*. It has been hypothesized that, in autogamous species, positive epistatic effects due to coadapted gene complexes can be perpetuated easily, compared with allogamous species (Allard, 1988; Garcia et al., 2008; Shi et al., 2011). As both *B. juncea* (5–20%) and *B. napus* (10–30%) are partially allogamous crops (Asthana and Singh, 1973; Rakow and Woods, 1987), the important role played by epistasis in regulating the genetic mechanisms underlying heterosis appears to be similar (Radoev et al., 2008; Basunanda et al., 2010; Shi et al., 2011; Li et al., 2012). The fact that, in this study, most of the epistasis occurred between complementary loci without detectable main effects is found to be in sharp agreement with the results reported by Radoev et al. (2008), Basunanda et al. (2010), and Shi et al. (2011).

A number of loci involved in epistasis were found to be interacting with more than one locus. For example, in BC-V population, the locus on A7 involved in plant height interacted with loci on B1 and B6. Such interactions were also observed where one locus interacted with two different loci, which were detected in different data sets. For example, a locus detected on linkage group B1 (39.48 cM to 40.7 cM) interacted with a locus on linkage group B6 (59.85 cM to 60.08 cM) detected in BC-E and also with a locus on linkage group B2 (167.87 cM to 170.43 cM) detected in MPH-V. This participation of loci in multi-locus interactions has been hypothesized to constitute higher order epistatic interactions (Zhao et al., 2005; Radoev et al., 2008). Similar results were observed by Shang et al. (2016) in upland cotton, where they found E-QTL, showing pleiotropic effects.

This research revealed that the genetic basis of heterosis of yield-influencing traits in *B. juncea* is complicated but definitely involves a large number of loci-exhibiting cumulative effects of dominance, overdominance, and a large number of epistatic interactions. The QTL detected in multiple environments can be considered as candidate loci for further studies. This study will provide the essential stimulus for further research on heterosis studies on *B. juncea*, which can be performed by evaluating testcrosses with related or unrelated testers to uncover stable QTL in inbred lines and across testers. The selection of these consistent QTL across testers can lead to better yield performance and in the development of hybrid combinations. The common QTLs validated across multiple trials and multiple populations will provide a valuable resource for MAS and the further research.

## Data Availability Statement

The original contributions presented in the study are included in the article/Supplementary Material, further inquiries can be directed to the corresponding author/s.

## Author Contributions

Aakanksha, and SY carried out the phenotyping and mapping work. BY helped in field experiments and candidate gene analysis. VG helped with genotyping. AM developed microspore-derived F_1_DH mapping population. DP and AP conceived and supervised the overall study and along with Aakanksha and SY wrote the manuscript. All authors have read and approved the final manuscript.

## Funding

This study was funded by the Department of Biotechnology (DBT), Government of India through the award of two projects—(1) Centre of Excellence on Genome Mapping and Molecular Breeding of Brassicas (BT/01/COE/08/06/-II) and (2) DBTUDSC Partnership Centre on Genetic Manipulation of Brassicas (BT/01/NDDB/UDSC/2016).

## Conflict of Interest

The authors declare that the research was conducted in the absence of any commercial or financial relationships that could be construed as a potential conflict of interest.

## Publisher's Note

All claims expressed in this article are solely those of the authors and do not necessarily represent those of their affiliated organizations, or those of the publisher, the editors and the reviewers. Any product that may be evaluated in this article, or claim that may be made by its manufacturer, is not guaranteed or endorsed by the publisher.
