# Supplementary material for: Genetic Analysis of Heterosis for Yield Influencing Traits in Brassica juncea Using a Doubled Haploid Population and Its Backcross Progenies
Source: Front Plant Sci. 2021 Sep 16;12:721631. doi: 10.3389/fpls.2021.721631 (PMC8481694; doi:10.3389/fpls.2021.721631)
Supplement: Supplementary file 1 [file Table_1.DOCX]

**Supplementary Table S1** Field Experimental conditions in which the parental lines Varuna, EH-2, F_1_, VEH doubled haploid lines and two backcross populations BC-V and BC-E were evaluated

| **Experimental Field Location** | **Growing season** | **Latitude, Longitude** | **Altitude (m)** | **Year** | **Month** | **Average Temperature (°C)** | **Precipitation (mm)** |
| --- | --- | --- | --- | --- | --- | --- | --- |
| Delhi (India) | 2014-15 | 28°45' N, 76°58' E | 216 | 2014 | Oct | 27.0 | 0 |
|  |  |  |  | 2014 | Nov | 20.0 | 0 |
|  |  |  |  | 2014 | Dec | 13.8 | 0 |
|  |  |  |  | 2015 | Jan | 12.4 | 0 |
|  |  |  |  | 2015 | Feb | 19.0 | 0 |
|  |  |  |  | 2015 | Mar | 22.1 | 0 |
|  |  |  |  | 2015 | Apr | 27.8 | 0 |
|  |  |  |  | 2015 | May | 33.8 | 0 |
|  | | | | | | | |
| Delhi (India) | 2015-16 | 28°45' N, 76°58' E | 216 | 2015 | Oct | 26.8 | 0 |
|  |  |  |  | 2015 | Nov | 20.9 | 0 |
|  |  |  |  | 2015 | Dec | 15.0 | 0 |
|  |  |  |  | 2016 | Jan | 14.6 | 0 |
|  |  |  |  | 2016 | Feb | 17.8 | 0 |
|  |  |  |  | 2016 | Mar | 24.0 | 0 |
|  |  |  |  | 2016 | Apr | 30.5 | 0 |
|  |  |  |  | 2016 | May | 32.9 | 0 |
|  | | | | | | | |
| Delhi (India) | 2016-17 | 28°45' N, 77°6' E | 216 | 2016 | Oct | 26.3 | 0 |
|  |  |  |  | 2016 | Nov | 20.2 | 0 |
|  |  |  |  | 2016 | Dec | 15.5 | 0 |
|  |  |  |  | 2017 | Jan | 14.3 | 0 |
|  |  |  |  | 2017 | Feb | 18.2 | 0 |
|  |  |  |  | 2017 | Mar | 23.3 | 0 |
|  |  |  |  | 2017 | Apr | 30.6 | 0 |
|  |  |  |  | 2017 | May | 32.6 | 0 |
