# Supplementary material for: Genetic Analysis of Heterosis for Yield Influencing Traits in Brassica juncea Using a Doubled Haploid Population and Its Backcross Progenies
Source: Front Plant Sci. 2021 Sep 16;12:721631. doi: 10.3389/fpls.2021.721631 (PMC8481694; doi:10.3389/fpls.2021.721631)
Supplement: Supplementary file 3 [file Table_3.DOCX]

**Supplementary Table S3.** Summary statistics of plant architectural traits (plant height, main shoot length, number of primary branches and number of secondary branches) and days to flowering in VEH, BC-V, BC-E, MPH-V, and MPH-E

| **Trait** | **Trial** | **Mean** | | | | | **Min** | | | | | **Max** | | | | | **Parents** | | **F1** | **MPH %** |
| --- | --- | --- | --- | --- | --- | --- | --- | --- | --- | --- | --- | --- | --- | --- | --- | --- | --- | --- | --- | --- |
|  |  | **VEH** | **BC-V** | **BC-E** | **MPH-V**  **(%)** | **MPH-E**  **(%)** | **VEH** | **BC-V** | **BC-E** | **MPH-V**  **(%)** | **MPH-E**  **(%)** | **VEH** | **BC-V** | **BC-E** | **MPH-V**  **(%)** | **MPH-E**  **(%)** | **EH-2** | **Varuna** |  |  |
| **PH** | **2014-15** | 213.18 | 214.07 | 231.59 | 6.00 | 6.40 | 152.07 | 184.73 | 185.60 | -8.98 | -7.17 | 274.62 | 253.27 | 282.73 | 38.72 | 41.93 | 222.13 | 190.73 | 226.87 | 9.9** |
|  | **2015-16** | 208.60 | 211.39 | 219.91 | 5.33 | 2.48 | 147.44 | 161.78 | 182.44 | -16.56 | -3.17 | 262.89 | 257.22 | 252.78 | 40.22 | 46.06 | 220.56 | 192.78 | 226.89 | 9.8** |
|  | **2016-17** | 218.40 | 208.92 | 230.92 | 3.07 | 4.83 | 159.60 | 175.07 | 193.23 | -19.38 | -12.14 | 283.33 | 256.27 | 270.57 | 34.87 | 27.83 | 222.15 | 187.00 | 217.73 | 6.4** |
|  |  |  |  |  |  |  |  |  |  |  |  |  |  |  |  |  |  |  |  |  |
| **DF** | **2014-15** | 57.00 | 55.15 | 53.80 | 0.27 | 1.51 | 40.67 | 46.00 | 44.67 | -12.00 | -8.83 | 93.00 | 75.00 | 70.67 | 20.17 | 10.00 | 49.00 | 53.00 | 57.67 | 13.10 |
|  | **2015-16** | 64.63 | 64.82 | 63.14 | 4.58 | 8.27 | 45.67 | 51.67 | 50.00 | -7.67 | -4.17 | 81.67 | 75.00 | 75.33 | 13.67 | 14.00 | 52.00 | 59.33 | 60.33 | 8.40 |
|  | **2016-17** | 56.25 | 54.28 | 55.86 | 2.49 | 2.57 | 43.33 | 45.33 | 45.67 | -12.17 | -5.17 | 79.67 | 66.00 | 67.00 | 8.17 | 8.00 | 52.67 | 49.67 | 55.00 | 7.5* |
|  |  |  |  |  |  |  |  |  |  |  |  |  |  |  |  |  |  |  |  |  |
| **MSL** | **2014-15** | 66.68 | 68.10 | 64.70 | 7.46 | -6.33 | 37.48 | 48.83 | 44.80 | -6.67 | 15.77 | 100.53 | 90.76 | 83.67 | 22.51 | 15.77 | 71.47 | 60.07 | 58.73 | -10.70 |
|  | **2015-16** | 72.28 | 72.97 | 70.29 | 1.31 | -5.90 | 46.33 | 55.22 | 55.67 | -15.28 | 7.83 | 102.56 | 90.11 | 87.44 | 20.44 | 7.83 | 77.11 | 71.78 | 67.11 | -9.90 |
|  | **2016-17** | 70.22 | 77.00 | 68.67 | 5.01 | -3.55 | 42.33 | 56.13 | 52.67 | -25.53 | 13.24 | 103.27 | 97.60 | 91.07 | 21.70 | 13.24 | 72.18 | 76.43 | 68.47 | -7.9* |
|  |  |  |  |  |  |  |  |  |  |  |  |  |  |  |  |  |  |  |  |  |
| **PBR** | **2014-15** | 6.49 | 7.11 | 7.44 | 13.85 | 11.96 | 2.87 | 3.73 | 4.73 | -2.23 | -1.99 | 11.02 | 9.53 | 9.40 | 2.83 | 2.05 | 6.80 | 6.00 | 7.10 | 10.90 |
|  | **2015-16** | 5.71 | 5.87 | 7.63 | 0.26 | 39.62 | 3.56 | 4.11 | 5.67 | -2.44 | 0.33 | 7.89 | 7.33 | 9.33 | 1.39 | 4.11 | 5.22 | 6.00 | 7.22 | 28.7** |
|  | **2016-17** | 5.54 | 4.82 | 6.37 | -0.31 | 8.24 | 2.80 | 2.73 | 4.40 | -1.53 | -1.22 | 8.80 | 6.87 | 8.27 | 1.70 | 2.15 | 6.23 | 4.13 | 6.27 | 20.9** |

* significant at P-value ≤ 0.05

**significant at P-value ≤ 0.01

**Supplementary Table S3. (continued..)** Summary statistics of silique related traits (single plant yield, silique density, siliqus length, number of silique on a main shoot, number of silique on a plant and seeds per silique) in VEH, BC-V, BC-E, MPH-V, and MPH-E

| **Trait** | **Trial** | **Mean** | | | | | **Min** | | | | | **Max** | | | | | **Parents** | | **F1** | **MPH %** |
| --- | --- | --- | --- | --- | --- | --- | --- | --- | --- | --- | --- | --- | --- | --- | --- | --- | --- | --- | --- | --- |
|  |  | **VEH** | **BC-V** | **BC-E** | **MPH-V**  **(%)** | **MPH-E**  **(%)** | **VEH** | **BC-V** | **BC-E** | **MPH-V**  **(%)** | **MPH-E**  **(%)** | **VEH** | **BC-V** | **BC-E** | **MPH-V**  **(%)** | **MPH-E**  **(%)** | **EH-2** | **Varuna** |  |  |
| **SBR** | 2014-15 | 13.08 | 14.20 | 12.45 | 30.10 | -4.05 | 4.87 | 7.47 | 7.80 | -4.13 | -6.50 | 26.47 | 23.13 | 19.13 | 11.68 | 5.03 | 12.87 | 8.75 | 12.07 | 11.60 |
|  | 2015-16 | 8.62 | 10.30 | 10.44 | 27.32 | 23.19 | 3.06 | 4.83 | 5.44 | -3.83 | -2.50 | 14.33 | 18.89 | 16.22 | 12.00 | 7.56 | 8.33 | 7.56 | 12.22 | 53.8** |
|  | 2016-17 | 9.92 | 7.01 | 11.10 | -17.77 | 9.52 | 3.07 | 3.33 | 6.73 | -8.07 | -4.78 | 20.07 | 11.93 | 16.88 | 4.07 | 5.33 | 10.35 | 7.13 | 13.00 | 48.7** |
|  |  |  |  |  |  |  |  |  |  |  |  |  |  |  |  |  |  |  |  |  |
| **SPY** | 2014-15 | 9.46 | 14.45 | 14.60 | 61.09 | 12.22 | 1.57 | 8.08 | 6.38 | -3.41 | -5.59 | 20.73 | 26.19 | 26.46 | 18.04 | 12.75 | 16.56 | 8.48 | 17.56 | 40.3** |
|  | 2015-16 | 9.88 | 14.30 | 12.25 | 45.25 | 13.48 | 1.20 | 6.88 | 5.08 | -3.50 | -2.99 | 18.71 | 25.40 | 22.58 | 13.19 | 15.25 | 11.71 | 9.81 | 16.68 | 55.0** |
|  | 2016-17 | 12.78 | 16.42 | 15.12 | 33.50 | 5.62 | 4.34 | 8.62 | 6.69 | -8.19 | -4.83 | 25.49 | 23.48 | 27.83 | 12.64 | 12.54 | 15.85 | 11.82 | 23.05 | 66.6** |
|  |  |  |  |  |  |  |  |  |  |  |  |  |  |  |  |  |  |  |  |  |
| **SQD** | 2014-15 | 0.87 | 0.82 | 0.92 | 0.61 | 1.10 | 0.52 | 0.58 | 0.69 | -0.21 | -0.17 | 1.31 | 1.10 | 1.27 | 0.27 | 0.41 | 0.95 | 0.76 | 1.05 | 22.6** |
|  | 2015-16 | 0.82 | 0.75 | 0.92 | 0.67 | 21.05 | 0.54 | 0.55 | 0.74 | -0.17 | -0.04 | 1.16 | 0.93 | 1.08 | 0.16 | 0.31 | 0.70 | 0.67 | 0.89 | 29.0** |
|  | 2016-17 | 0.84 | 0.72 | 0.91 | -2.70 | 7.06 | 0.51 | 0.51 | 0.62 | -0.26 | -0.18 | 1.26 | 1.02 | 1.29 | 0.39 | 0.33 | 0.86 | 0.64 | 0.86 | 15.1* |
|  |  |  |  |  |  |  |  |  |  |  |  |  |  |  |  |  |  |  |  |  |
| **SQL** | 2014-15 | 3.77 | 4.43 | 3.32 | 5.48 | -3.07 | 2.77 | 3.68 | 2.67 | -0.36 | -0.45 | 5.05 | 5.41 | 4.33 | 0.92 | 0.43 | 3.08 | 4.63 | 4.01 | 3.90 |
|  | 2015-16 | 4.43 | 5.24 | 3.88 | 6.83 | -1.65 | 3.07 | 4.54 | 3.27 | -0.63 | -0.64 | 5.88 | 6.07 | 4.56 | 0.62 | 0.55 | 3.46 | 5.38 | 4.52 | 2.40 |
|  | 2016-17 | 4.18 | 5.01 | 3.82 | 1.83 | -1.80 | 2.85 | 3.77 | 3.03 | -1.14 | -0.62 | 5.82 | 6.13 | 7.50 | 0.77 | 0.48 | 3.60 | 5.66 | 4.76 | 2.9* |
|  |  |  |  |  |  |  |  |  |  |  |  |  |  |  |  |  |  |  |  |  |
| **SQMS** | 2014-15 | 56.57 | 55.28 | 59.13 | 8.21 | -4.97 | 35.40 | 40.93 | 42.75 | -8.77 | -16.00 | 79.07 | 67.73 | 74.67 | 19.33 | 12.10 | 67.87 | 45.60 | 61.53 | 8.50 |
|  | 2015-16 | 58.86 | 54.79 | 64.54 | 2.23 | 14.04 | 35.00 | 41.00 | 52.22 | -9.78 | -10.17 | 86.00 | 66.89 | 78.56 | 16.22 | 24.39 | 54.33 | 48.33 | 59.67 | 16.2* |
|  | 2016-17 | 57.44 | 54.54 | 61.71 | 2.74 | 3.36 | 34.27 | 43.80 | 45.73 | -9.87 | -16.87 | 78.00 | 66.87 | 78.63 | 15.83 | 25.50 | 61.97 | 48.73 | 58.93 | 6.50 |
|  |  |  |  |  |  |  |  |  |  |  |  |  |  |  |  |  |  |  |  |  |
| **SQPL** | 2014-15 | 460.92 | 459.17 | 602.15 | 28.35 | 6.12 | 198.67 | 279.67 | 371.33 | -90.93 | -188.83 | 835.77 | 695.53 | 971.14 | 363.13 | 389.21 | 673.93 | 254.60 | 478.67 | 3.10 |
|  | 2015-16 | 426.33 | 354.06 | 509.95 | -0.39 | 25.81 | 243.67 | 222.67 | 346.78 | -198.56 | -74.28 | 744.17 | 630.56 | 761.56 | 321.44 | 374.72 | 384.33 | 284.56 | 511.44 | 52.9** |
|  | 2016-17 | 417.14 | 370.91 | 543.55 | 11.53 | 7.91 | 182.09 | 220.20 | 385.33 | -100.58 | -102.02 | 1113.64 | 585.73 | 793.88 | 235.20 | 261.95 | 590.30 | 248.00 | 489.53 | 16.8* |
|  |  |  |  |  |  |  |  |  |  |  |  |  |  |  |  |  |  |  |  |  |
| **SSQ** | 2014-15 | 14.99 | 15.27 | 17.07 | 10.69 | 5.40 | 9.43 | 12.33 | 13.93 | -0.93 | -1.54 | 19.33 | 17.33 | 20.40 | 4.74 | 3.74 | 17.40 | 12.60 | 15.87 | 5.80 |
|  | 2015-16 | 15.49 | 14.94 | 16.58 | 3.28 | -1.31 | 11.56 | 12.44 | 13.89 | -1.61 | -3.22 | 19.22 | 18.22 | 19.44 | 3.17 | 2.61 | 18.11 | 13.44 | 16.44 | 4.20 |
|  | 2016-17 | 15.54 | 15.36 | 16.55 | 5.42 | -4.17 | 11.73 | 13.40 | 13.47 | -1.18 | -3.68 | 19.53 | 18.60 | 19.60 | 3.47 | 2.75 | 19.00 | 13.60 | 17.20 | 5.50 |

* significant at P-value ≤ 0.05

**significant at P-value ≤ 0.01

**Supplementary Table S3. (continued..)** Summary statistics of seed related traits (oil content, protein content and thousand seed weight) in VEH, BC-V, BC-E, MPH-V, and MPH-E

| **Trait** | **Trial** | **Mean** | | | | | **Min** | | | | | **Max** | | | | | **Parents** | | **F1** | **MPH %** |
| --- | --- | --- | --- | --- | --- | --- | --- | --- | --- | --- | --- | --- | --- | --- | --- | --- | --- | --- | --- | --- |
|  |  | **VEH** | **BC-V** | **BC-E** | **MPH-V**  **(%)** | **MPH-E**  **(%)** | **VEH** | **BC-V** | **BC-E** | **MPH-V**  **(%)** | **MPH-E**  **(%)** | **VEH** | **BC-V** | **BC-E** | **MPH-V**  **(%)** | **MPH-E**  **(%)** | **EH-2** | **Varuna** |  |  |
| **OIL** | **2014-15** | 37.52 | 39.52 | 37.81 | 5.33 | 3.33 | 31.29 | 35.99 | 32.30 | -1.42 | -2.15 | 43.89 | 42.96 | 41.86 | 5.57 | 4.20 | 35.66 | 37.52 | 40.40 | 10.4* |
|  | **2015-16** | 36.90 | 39.23 | 37.66 | 3.96 | 3.99 | 30.10 | 34.32 | 32.47 | -2.73 | -2.38 | 42.42 | 42.53 | 41.95 | 4.39 | 5.78 | 35.53 | 38.57 | 40.82 | 10.2** |
|  | **2016-17** | 37.77 | 40.76 | 37.93 | 3.22 | 2.72 | 29.56 | 37.28 | 32.63 | -2.72 | -2.31 | 43.07 | 43.54 | 41.81 | 3.78 | 4.59 | 36.08 | 41.21 | 40.50 | 4.80 |
|  |  |  |  |  |  |  |  |  |  |  |  |  |  |  |  |  |  |  |  |  |
| **PRO** | **2014-15** | 25.28 | 23.40 | 25.33 | -3.31 | -1.32 | 21.56 | 20.84 | 23.37 | -3.03 | -2.45 | 29.27 | 25.42 | 28.94 | 1.59 | 2.01 | 26.06 | 23.12 | 23.83 | -3.10 |
|  | **2015-16** | 26.41 | 24.51 | 26.53 | -4.35 | -4.29 | 22.73 | 22.36 | 23.96 | -3.31 | -3.34 | 30.66 | 26.51 | 29.07 | 0.99 | 1.45 | 29.03 | 24.84 | 24.72 | -8.2* |
|  | **2016-17** | 26.27 | 23.88 | 26.64 | -3.36 | -1.88 | 21.28 | 22.12 | 23.62 | -3.11 | -3.14 | 30.31 | 25.69 | 28.65 | 1.43 | 1.28 | 28.03 | 23.15 | 24.73 | -3.40 |
|  |  |  |  |  |  |  |  |  |  |  |  |  |  |  |  |  |  |  |  |  |
| **TSW** | **2014-15** | 2.73 | 3.53 | 2.48 | 4.13 | -1.00 | 1.70 | 2.78 | 1.91 | -0.52 | -0.65 | 4.30 | 4.67 | 3.08 | 0.92 | 0.39 | 2.28 | 4.05 | 2.79 | -12.00 |
|  | **2015-16** | 3.00 | 3.96 | 2.74 | -4.12 | 0.37 | 1.63 | 2.92 | 2.11 | -1.03 | -0.56 | 4.50 | 5.30 | 3.37 | 0.79 | 0.78 | 2.46 | 5.26 | 3.36 | -12.9* |
|  | **2016-17** | 3.07 | 3.98 | 2.78 | -1.12 | -0.54 | 1.85 | 2.84 | 2.12 | -3.61 | -0.57 | 5.02 | 5.11 | 3.74 | 0.73 | 0.66 | 2.52 | 4.98 | 3.48 | -7.30 |

* significant at P-value ≤ 0.05

**significant at P-value ≤ 0.01

**Supplementary Figure S1. High density linkage map of VEH population constructed with IP, SSR, KASP-SNP and Chip based markers**

**Supplementary Figure S1. Continued**

**Supplementary Figure S1. Continued**

**Supplementary Figure S1. Continued**

**Supplementary Figure S1. Continued**
