# Supplementary material for: Genetic Analysis of Heterosis for Yield Influencing Traits in Brassica juncea Using a Doubled Haploid Population and Its Backcross Progenies
Source: Front Plant Sci. 2021 Sep 16;12:721631. doi: 10.3389/fpls.2021.721631 (PMC8481694; doi:10.3389/fpls.2021.721631)
Supplement: Supplementary file 4 [file Table_4.DOCX]

**Supplementary Table S4.** Genetic variance of 14 yield influencing traits in the three mapping populations in the three crop growing seasons

| **Population** | **Year** | **PH** | **DF** | **MSL** | **PBR** | **SBR** | **SPY** | **SQD** | **SQL** | **SQMS** | **SQPL** | **SSQ** | **OIL** | **PRO** | **TSW** |
| --- | --- | --- | --- | --- | --- | --- | --- | --- | --- | --- | --- | --- | --- | --- | --- |
| **VEH** | **2014-15** | 754.4 | 103.6 | 147.4 | 1.74 | 11.28 | 7.06 | 0.02 | 0.23 | 51.76 | 9708 | 3.01 | 6.25 | 1.76 | 0.19 |
|  | **2015-16** | 553.4 | 54.27 | 68.29 | 0.41 | 2.71 | 3.94 | 0.01 | 0.2 | 32.94 | 2499 | 1.56 | 5.52 | 1.13 | 0.24 |
|  | **2016-17** | 757.5 | 80.99 | 137 | 1.51 | 8.07 | 9.14 | 0.02 | 0.27 | 30.65 | 8536 | 2.41 | 5.71 | 1.43 | 0.25 |
| **BC-V**  **(VEH X VARUNA)** | **2014-15** | 228.3 | 28.68 | 46.89 | 0.97 | 5.58 | 4.06 | 0.01 | 0.05 | 18.37 | 2922 | 0.61 | 1.28 | 0.29 | 0.1 |
|  | **2015-16** | 247.6 | 16.08 | 28.47 | 0.12 | 1.51 | 1.93 | 0 | 0.07 | 16.86 | 876.4 | 0.51 | 1.24 | 0.27 | 0.14 |
|  | **2016-17** | 231.4 | 23.34 | 69.96 | 0.83 | 1.35 | 4.47 | 0.01 | 0.08 | 16.8 | 2385 | 0.57 | 0.93 | 0.33 | 0.1 |
| **BC-E**  **(VEH X EH2)** | **2014-15** | 239.5 | 24.06 | 35.71 | 0.47 | 1.37 | 3.97 | 0.01 | 0.05 | 10.35 | 4646 | 0.71 | 1.93 | 0.43 | 0.03 |
|  | **2015-16** | 233.6 | 22.53 | 29.51 | 0.33 | 1.34 | 2.27 | 0 | 0.05 | 11.89 | 1254 | 0.64 | 2.42 | 0.49 | 0.05 |
|  | **2016-17** | 241.5 | 25.92 | 54.02 | 0.8 | 1.28 | 5.6 | 0.01 | 0.06 | 16.19 | 1323 | 0.88 | 1.92 | 0.31 | 0.06 |

**Supplementary Table S4. (continued..)** Heritability of 14 yield influencing traits in the three mapping populations in the three crop growing seasons

| **Population** | **Year** | **PH** | **DF** | **MSL** | **PBR** | **SBR** | **SPY** | **SQD** | **SQL** | **SQMS** | **SQPL** | **SSQ** | **OIL** | **PRO** | **TSW** |
| --- | --- | --- | --- | --- | --- | --- | --- | --- | --- | --- | --- | --- | --- | --- | --- |
| **VEH** | **2014-15** | 0.84 | 0.81 | 0.73 | 0.58 | 0.4 | 0.35 | 0.64 | 0.77 | 0.42 | 0.35 | 0.59 | 0.76 | 0.63 | 0.71 |
|  | **2015-16** | 0.8 | 0.7 | 0.52 | 0.34 | 0.26 | 0.18 | 0.57 | 0.76 | 0.36 | 0.14 | 0.49 | 0.62 | 0.44 | 0.77 |
|  | **2016-17** | 0.87 | 0.88 | 0.77 | 0.71 | 0.51 | 0.45 | 0.52 | 0.86 | 0.27 | 0.44 | 0.67 | 0.69 | 0.54 | 0.76 |
| **BC-V**  **(VEH X VARUNA)** | **2014-15** | 0.67 | 0.51 | 0.59 | 0.57 | 0.32 | 0.16 | 0.58 | 0.52 | 0.34 | 0.19 | 0.38 | 0.4 | 0.2 | 0.58 |
|  | **2015-16** | 0.69 | 0.53 | 0.36 | 0.16 | 0.14 | 0.08 | 0.52 | 0.61 | 0.33 | 0.09 | 0.32 | 0.29 | 0.18 | 0.58 |
|  | **2016-17** | 0.75 | 0.71 | 0.66 | 0.68 | 0.36 | 0.27 | 0.66 | 0.7 | 0.43 | 0.33 | 0.4 | 0.36 | 0.26 | 0.46 |
| **BC-E**  **(VEH X EH2)** | **2014-15** | 0.63 | 0.65 | 0.46 | 0.41 | 0.13 | 0.16 | 0.4 | 0.59 | 0.16 | 0.18 | 0.34 | 0.53 | 0.3 | 0.4 |
|  | **2015-16** | 0.63 | 0.53 | 0.42 | 0.27 | 0.11 | 0.1 | 0.24 | 0.63 | 0.2 | 0.06 | 0.3 | 0.53 | 0.23 | 0.52 |
|  | **2016-17** | 0.71 | 0.79 | 0.68 | 0.57 | 0.21 | 0.3 | 0.58 | 0.71 | 0.26 | 0.11 | 0.51 | 0.58 | 0.19 | 0.65 |
