# Supplementary material for: Genetic Analysis of Heterosis for Yield Influencing Traits in Brassica juncea Using a Doubled Haploid Population and Its Backcross Progenies
Source: Front Plant Sci. 2021 Sep 16;12:721631. doi: 10.3389/fpls.2021.721631 (PMC8481694; doi:10.3389/fpls.2021.721631)
Supplement: Supplementary file 6 [file Table_6.DOCX]

**Supplementary Table S6.** Coefficients of correlation for the plant architectural traits and days to flowering between the VEH lines, Backcross performance and Mid-parent heterosis for the 14 traits measured in three crop seasons (2014-15, 2015-16 and 2016-17)

| **Plant architectural traits and Days to flowering** | | | | | | | | | | | | | | | | |
| --- | --- | --- | --- | --- | --- | --- | --- | --- | --- | --- | --- | --- | --- | --- | --- | --- |
|  |  | **2014-15** | | | | | **2015-16** | | | | | **2016-17** | | | | |
| **Trait** | **Dataset** | **BC-V** | **BC-E** | **VEH** | **MPH-V** | **MPH-E** | **BC-V** | **BC-E** | **VEH** | **MPH-V** | **MPH-E** | **BC-V** | **BC-E** | **VEH** | **MPH-V** | **MPH-E** |
| **PH** | **BC-V** | 1 |  |  |  |  | 1 |  |  |  |  | 1 |  |  |  |  |
|  | **BC-E** | 0.759** | 1 |  |  |  | 0.765** | 1 |  |  |  | 0.784** | 1 |  |  |  |
|  | **VEH** | 0.833** | 0.837** | 1 |  |  | 0.834** | 0.810** | 1 |  |  | 0.837** | 0.882** | 1 |  |  |
|  | **MPH-V** | 0.505** | 0.064 | -0.057 | 1 |  | 0.702** | 0.319** | 0.193* | 1 |  | 0.488** | 0.023 | -0.069 | 1 |  |
|  | **MPH-E** | 0.12 | 0.554** | 0.008 | 0.205** | 1 | 0.282** | 0.702** | 0.160* | 0.296** | 1 | 0.159 | 0.534** | 0.073 | 0.173* | 1 |
| **DF** | **BC-V** | 1 |  |  |  |  | 1 |  |  |  |  | 1 |  |  |  |  |
|  | **BC-E** | 0.711** | 1 |  |  |  | 0.611** | 1 |  |  |  | 0.770** | 1 |  |  |  |
|  | **VEH** | 0.798** | 0.857** | 1 |  |  | 0.724** | 0.734** | 1 |  |  | 0.822** | 0.861** | 1 |  |  |
|  | **MPH-V** | 0.521** | -0.043 | -0.104 | 1 |  | 0.538** | -0.029 | -0.195* | 1 |  | 0.464** | 0.053 | -0.078 | 1 |  |
|  | **MPH-E** | -0.154 | 0.283** | -0.257** | 0.111 | 1 | 0.121 | 0.686** | 0.014 | 0.151 | 1 | 0.117 | 0.505** | -0.005 | 0.233** | 1 |
| **MSL** | **BC-V** | 1 |  |  |  |  | 1 |  |  |  |  | 1 |  |  |  |  |
|  | **BC-E** | 0.592** | 1 |  |  |  | 0.383** | 1 |  |  |  | 0.590** | 1 |  |  |  |
|  | **VEH** | 0.655** | 0.698** | 1 |  |  | 0.484** | 0.577** | 1 |  |  | 0.647** | 0.702** | 1 |  |  |
|  | **MPH-V** | 0.574** | 0.003 | -0.243** | 1 |  | 0.720** | -0.036 | -0.263** | 1 |  | 0.740** | 0.15 | -0.034 | 1 |  |
|  | **MPH-E** | -0.008 | 0.486** | -0.287** | 0.301** | 1 | 0.045 | 0.706** | -0.171* | 0.185* | 1 | 0.127 | 0.639** | -0.099 | 0.253** | 1 |
| **PBR** | **BC-V** | 1 |  |  |  |  | 1 |  |  |  |  | 1 |  |  |  |  |
|  | **BC-E** | 0.436** | 1 |  |  |  | 0.283** | 1 |  |  |  | 0.578** | 1 |  |  |  |
|  | **VEH** | 0.699** | 0.540** | 1 |  |  | 0.375** | 0.359** | 1 |  |  | 0.756** | 0.700** | 1 |  |  |
|  | **MPH-V** | 0.746** | 0.106 | 0.045 | 1 |  | 0.745** | 0.031 | -0.344** | 1 |  | 0.764** | 0.178* | 0.151 | 1 |  |
|  | **MPH-E** | -0.203** | 0.582** | -0.373** | 0.075 | 1 | 0.096 | 0.858** | -0.171* | 0.224** | 1 | 0.129 | 0.768** | 0.079 | 0.117 | 1 |
| **SBR** | **BC-V** | 1 |  |  |  |  | 1 |  |  |  |  | 1 |  |  |  |  |
|  | **BC-E** | 0.312** | 1 |  |  |  | 0.196* | 1 |  |  |  | 0.269** | 1 |  |  |  |
|  | **VEH** | 0.514** | 0.391** | 1 |  |  | 0.331** | 0.357** | 1 |  |  | 0.296** | 0.287** | 1 |  |  |
|  | **MPH-V** | 0.755** | 0.059 | -0.175* | 1 |  | 0.854** | -0.002 | -0.208** | 1 |  | 0.594** | -0.016 | -0.593** | 1 |  |
|  | **MPH-E** | -0.176* | 0.561** | -0.542** | 0.212** | 1 | 0.027 | 0.863** | -0.162* | 0.118 | 1 | 0.01 | 0.668** | -0.521** | 0.448** | 1 |

*-Significant at 5%, **- Significant at 1%

**Supplementary Table S6. (continued..)** Coefficients of correlation for silique related traits between the VEH lines, Backcross performance and Mid-parent heterosis for the 14 traits measured in three crop seasons (2014-15, 2015-16 and 2016-17)

| **Silique related traits** | | | | | | | | | | | | | | | | |
| --- | --- | --- | --- | --- | --- | --- | --- | --- | --- | --- | --- | --- | --- | --- | --- | --- |
|  |  | **2014-15** | | | | | **2015-16** | | | | | **2016-17** | | | | |
| **Trait** | **Dataset** | **BC-V** | **BC-E** | **VEH** | **MPH-V** | **MPH-E** | **BC-V** | **BC-E** | **VEH** | **MPH-V** | **MPH-E** | **BC-V** | **BC-E** | **VEH** | **MPH-V** | **MPH-E** |
| **SPY** | **BC-V** | 1 |  |  |  |  | 1 |  |  |  |  | 1 |  |  |  |  |
|  | **BC-E** | 0.113 | 1 |  |  |  | 0.164* | 1 |  |  |  | 0.253** | 1 |  |  |  |
|  | **VEH** | 0.184* | 0.301** | 1 |  |  | 0.260** | 0.225** | 1 |  |  | 0.237** | 0.305** | 1 |  |  |
|  | **MPH-V** | 0.875** | -0.04 | -0.315** | 1 |  | 0.865** | 0.047 | -0.259** | 1 |  | 0.810** | 0.056 | -0.378** | 1 |  |
|  | **MPH-E** | 0.018 | 0.865** | -0.218** | 0.125 | 1 | 0.024 | 0.860** | -0.304** | 0.181* | 1 | 0.115 | 0.830** | -0.278** | 0.278** | 1 |
| **SQD** | **BC-V** | 1 |  |  |  |  | 1 |  |  |  |  | 1 |  |  |  |  |
|  | **BC-E** | 0.465** | 1 |  |  |  | 0.581** | 1 |  |  |  | 0.452** | 1 |  |  |  |
|  | **VEH** | 0.642** | 0.633** | 1 |  |  | 0.638** | -0.262** | 1 |  |  | 0.546** | 0.567** | 1 |  |  |
|  | **MPH-V** | 0.647** | -0.039 | -0.181* | 1 |  | 0.587** | 0.993** | -0.262** | 1 |  | 0.731** | 0.059 | -0.181* | 1 |  |
|  | **MPH-E** | -0.027 | 0.674** | -0.159* | 0.123 | 1 | -0.053 | 0.285** | -0.328** | 0.280** | 1 | 0.16 | 0.812** | -0.02 | 0.205* | 1 |
| **SQL** | **BC-V** | 1 |  |  |  |  | 1 |  |  |  |  | 1 |  |  |  |  |
|  | **BC-E** | 0.574** | 1 |  |  |  | 0.690** | 1 |  |  |  | 0.364** | 1 |  |  |  |
|  | **VEH** | 0.677** | 0.797** | 1 |  |  | 0.721** | 0.767** | 1 |  |  | 0.706** | 0.478** | 1 |  |  |
|  | **MPH-V** | 0.433** | -0.252** | -0.370** | 1 |  | 0.614** | 0.134 | -0.104 | 1 |  | 0.552** | -0.073 | -0.201* | 1 |  |
|  | **MPH-E** | -0.156* | 0.326** | -0.310** | 0.184* | 1 | 0.051 | 0.440** | -0.247** | 0.355** | 1 | -0.092 | 0.350** | -0.376** | 0.313** | 1 |
| **SQMS** | **BC-V** | 1 |  |  |  |  | 1 |  |  |  |  | 1 |  |  |  |  |
|  | **BC-E** | 0.364** | 1 |  |  |  | 0.154* | 1 |  |  |  | 0.199* | 1 |  |  |  |
|  | **VEH** | 0.419** | 0.414** | 1 |  |  | 0.502** | 0.263** | 1 |  |  | 0.379** | 0.188* | 1 |  |  |
|  | **MPH-V** | 0.678** | 0.035 | -0.384** | 1 |  | 0.748** | -0.032 | -0.199* | 1 |  | 0.695** | 0.05 | -0.402** | 1 |  |
|  | **MPH-E** | 0.022 | 0.665** | -0.404** | 0.350** | 1 | -0.185* | 0.778** | -0.402** | 0.1 | 1 | -0.063 | 0.792** | -0.451** | 0.289** | 1 |
| **SQPL** | **BC-V** | 1 |  |  |  |  | 1 |  |  |  |  | 1 |  |  |  |  |
|  | **BC-E** | 0.073 | 1 |  |  |  | 0.091 | 1 |  |  |  | 0.109 | 1 |  |  |  |
|  | **VEH** | 0.375** | 0.222** | 1 |  |  | 0.208** | 0.238** | 1 |  |  | 0.381** | 0.241** | 1 |  |  |
|  | **MPH-V** | 0.717** | -0.094 | -0.378** | 1 |  | 0.777** | -0.07 | -0.453** | 1 |  | 0.727** | -0.071 | -0.358** | 1 |  |
|  | **MPH-E** | -0.156* | 0.810** | -0.392** | 0.139 | 1 | -0.014 | 0.875** | -0.262** | 0.156* | 1 | -0.133 | 0.797** | -0.394** | 0.161 | 1 |
| **SSQ** | **BC-V** | 1 |  |  |  |  | 1 |  |  |  |  | 1 |  |  |  |  |
|  | **BC-E** | 0.381** | 1 |  |  |  | 0.193* | 1 |  |  |  | 0.449** | 1 |  |  |  |
|  | **VEH** | 0.526** | 0.578** | 1 |  |  | 0.475** | 0.427** | 1 |  |  | 0.532** | 0.456** | 1 |  |  |
|  | **MPH-V** | 0.493** | -0.198* | -0.481** | 1 |  | 0.679** | -0.14 | -0.328** | 1 |  | 0.556** | 0.007 | -0.408** | 1 |  |
|  | **MPH-E** | -0.101 | 0.553** | -0.359** | 0.262** | 1 | -0.13 | 0.758** | -0.272** | 0.09 | 1 | -0.001 | 0.667** | -0.359** | 0.349** | 1 |

*-Significant at 5%, **- Significant at 1%

**Supplementary Table S6. (continued..)**Coefficients of correlation for seed related traits between the VEH lines, Backcross performance and Mid-parent heterosis for the 14 traits measured in three crop seasons (2014-15, 2015-16 and 2016-17)

| **Seed related traits** | | | | | | | | | | | | | | | | |
| --- | --- | --- | --- | --- | --- | --- | --- | --- | --- | --- | --- | --- | --- | --- | --- | --- |
|  |  | **2014-15** | | | | | **2015-16** | | | | | **2016-17** | | | | |
| **Trait** | **Dataset** | **BC-V** | **BC-E** | **VEH** | **MPH-V** | **MPH-E** | **BC-V** | **BC-E** | **VEH** | **MPH-V** | **MPH-E** | **BC-V** | **BC-E** | **VEH** | **MPH-V** | **MPH-E** |
| **OIL** | **BC-V** | 1 |  |  |  |  | 1 |  |  |  |  | 1 |  |  |  |  |
|  | **BC-E** | 0.423** | 1 |  |  |  | 0.537** | 1 |  |  |  | 0.408** | 1 |  |  |  |
|  | **VEH** | 0.624** | 0.717** | 1 |  |  | 0.610** | 0.652** | 1 |  |  | 0.590** | 0.594** | 1 |  |  |
|  | **MPH-V** | 0.481** | -0.305** | -0.385** | 1 |  | 0.561** | -0.038 | -0.314** | 1 |  | 0.451** | -0.181* | -0.455** | 1 |  |
|  | **MPH-E** | -0.135 | 0.571** | -0.163* | 0.023 | 1 | 0.118 | 0.684** | -0.106 | 0.252** | 1 | -0.018 | 0.629** | -0.205* | 0.227** | 1 |
| **PRO** | **BC-V** | 1 |  |  |  |  | 1 |  |  |  |  | 1 |  |  |  |  |
|  | **BC-E** | 0.369** | 1 |  |  |  | 0.331** | 1 |  |  |  | 0.270** | 1 |  |  |  |
|  | **VEH** | 0.514** | 0.504** | 1 |  |  | 0.401** | 0.422** | 1 |  |  | 0.368** | 0.522** | 1 |  |  |
|  | **MPH-V** | 0.585** | -0.081 | -0.395** | 1 |  | 0.688** | -0.001 | -0.389** | 1 |  | 0.674** | -0.156 | -0.440** | 1 |  |
|  | **MPH-E** | -0.061 | 0.632** | -0.351** | 0.267** | 1 | 0.082 | 0.785** | -0.231** | 0.265** | 1 | -0.022 | 0.673** | -0.280** | 0.212* | 1 |
| **TSW** | **BC-V** | 1 |  |  |  |  | 1 |  |  |  |  | 1 |  |  |  |  |
|  | **BC-E** | 0.496** | 1 |  |  |  | 0.571** | 1 |  |  |  | 0.434** | 1 |  |  |  |
|  | **VEH** | 0.669** | 0.658** | 1 |  |  | 0.659** | 0.577** | 1 |  |  | 0.500** | 0.668** | 1 |  |  |
|  | **MPH-V** | 0.750** | 0.081 | 0.01 | 1 |  | 0.780** | 0.278** | 0.043 | 1 |  | 0.751** | -0.012 | -0.197* | 1 |  |
|  | **MPH-E** | -0.262** | 0.335** | -0.489** | 0.082 | 1 | -0.104 | 0.448** | -0.471** | 0.253** | 1 | -0.039 | 0.483** | -0.330** | 0.207* | 1 |

*-Significant at 5%, **- Significant at 1%
