# Supplementary material for: Genetic Analysis of Heterosis for Yield Influencing Traits in Brassica juncea Using a Doubled Haploid Population and Its Backcross Progenies
Source: Front Plant Sci. 2021 Sep 16;12:721631. doi: 10.3389/fpls.2021.721631 (PMC8481694; doi:10.3389/fpls.2021.721631)
Supplement: Supplementary file 11 [file Data_Sheet_1.PDF]

A1

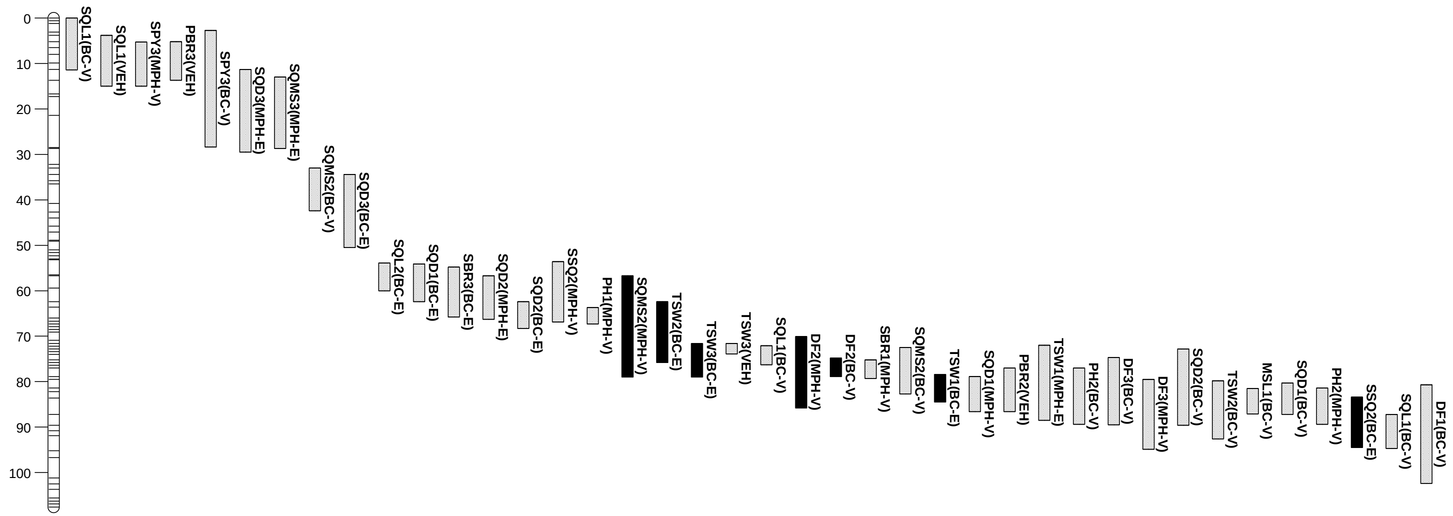

Supplementary Figure 1. The VEH map showing QTL for the 14 yield related traits detected in the five datasets (VEH, BC-V, BC-E, MPH-V and MPH-E). QTL are designated using the trait name initials followed by a digit identifying the trial number (1 for 2014-15; 2 for 2015-16; 3 for 2106-17) and the dataset name (in parenthesis) in which it was detected. Major QTL (phenotypic variance ≥ 10%) are shown with solid bars.

A2

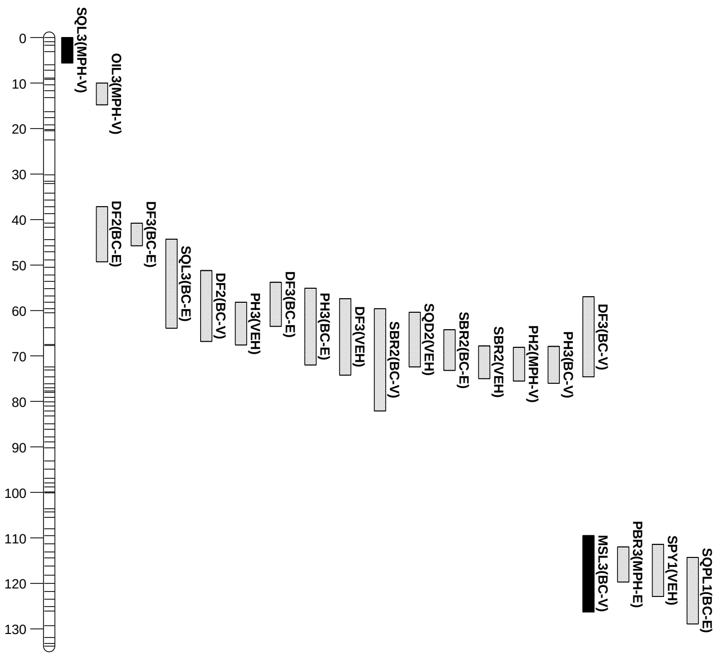
0
10
20
30
40
50
60
70
80
90
100
110
120
130
SQL3(MPH-V)
OIL3(MPH-V)
DF2(BC-E)
DF3(BC-E)
SQL3(BC-E)
DF2(BC-V)
PH3(VEH)
DF3(BC-E)
PH3(BC-E)
DF3(VEH)
SBR2(BC-V)
SQD2(VEH)
SBR2(BC-E)
SBR2(VEH)
PH2(MPH-V)
PH3(BC-V)
DF3(BC-V)
MSL3(BC-V)
PBR3(MPH-E)
SPY1(VEH)
SQPL1(BC-E)

A3

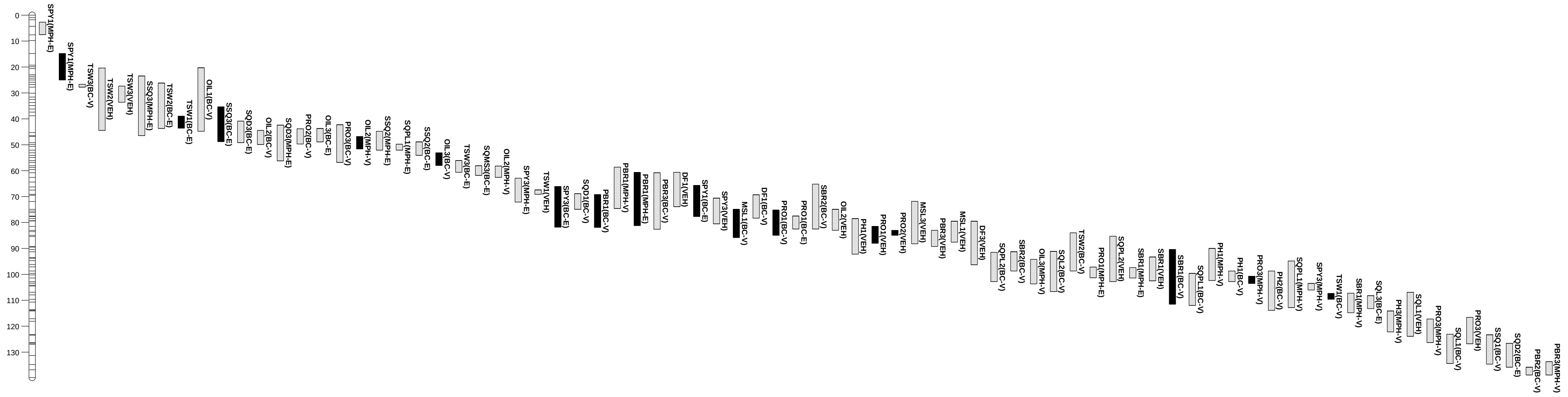

A4

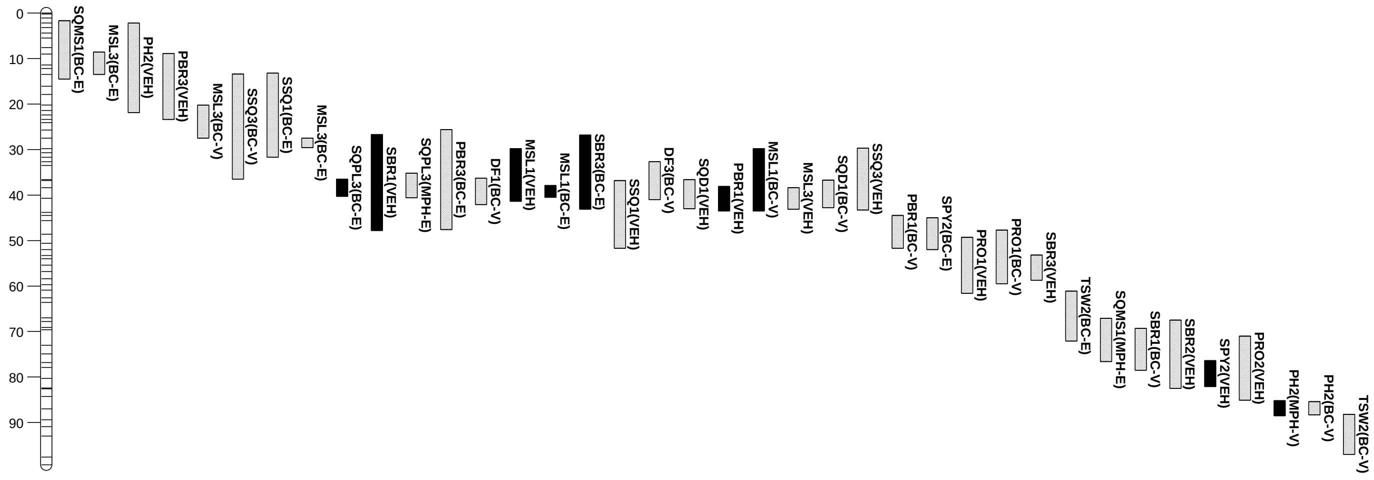
0
10
20
30
40
50
60
70
80
90
SQMS1(BC-E)
MSL3(BC-E)
PH2(VEH)
PBR3(VEH)
MSL3(BC-V)
SSQ3(BC-V)
SSQ1(BC-E)
MSL3(BC-E)
SQPL3(BC-E)
SBR1(VEH)
SQPL3(MPH-E)
PBR3(BC-E)
DF1(BC-V)
MSL1(VEH)
MSL1(BC-E)
SBR3(BC-E)
SSQ1(VEH)
DF3(BC-V)
SQD1(VEH)
PBR1(VEH)
MSL1(BC-V)
MSL3(VEH)
SQD1(BC-V)
SSQ3(VEH)
PBR1(BC-V)
SPY2(BC-E)
PRO1(VEH)
PRO1(BC-V)
SBR3(VEH)
TSW2(BC-E)
SQMS1(MPH-E)
SBR1(BC-V)
SBR2(VEH)
SPY2(VEH)
PRO2(VEH)
PH2(MPH-V)
PH2(BC-V)
TSW2(BC-V)

A5

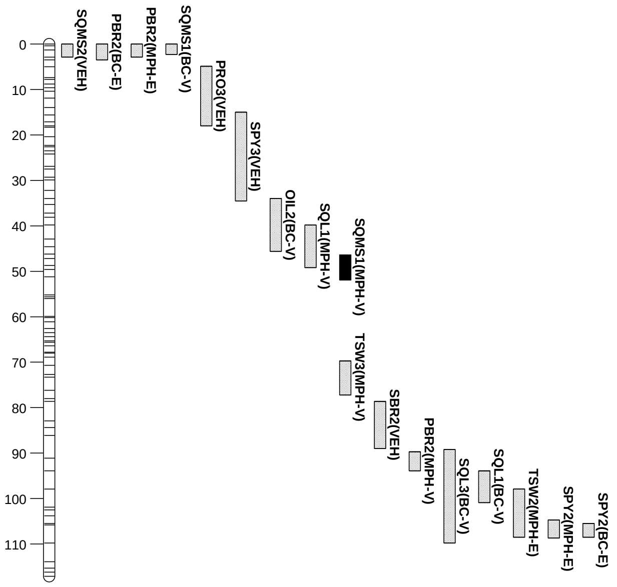

0
10
20
30
40
50
60
70
80
90
100
110
SQMS2(VEH)
PBR2(BC-E)
PBR2(MPH-E)
SQMS1(BC-V)
PRO3(VEH)
SPY3(VEH)
OIL2(BC-V)
SQL1(MPH-V)
SQMS1(MPH-V)
TSW3(MPH-V)
SBR2(VEH)
PBR2(MPH-V)
SQL3(BC-V)
SQL1(BC-V)
TSW2(MPH-E)
SPY2(MPH-E)
SPY2(BC-E)

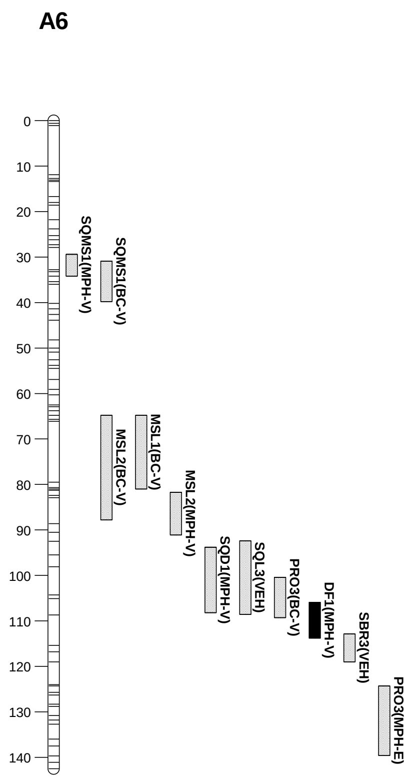
A6
0
10
20
30
40
50
60
70
80
90
100
110
120
130
140
SQMS1(MPH-V)
SQMS1(BC-V)
MSL2(BC-V)
MSL1(BC-V)
MSL2(MPH-V)
SQD1(MPH-V)
SQL3(VEH)
PRO3(BC-V)
DF1(MPH-V)
SBR3(VEH)
PRO3(MPH-E)

A7

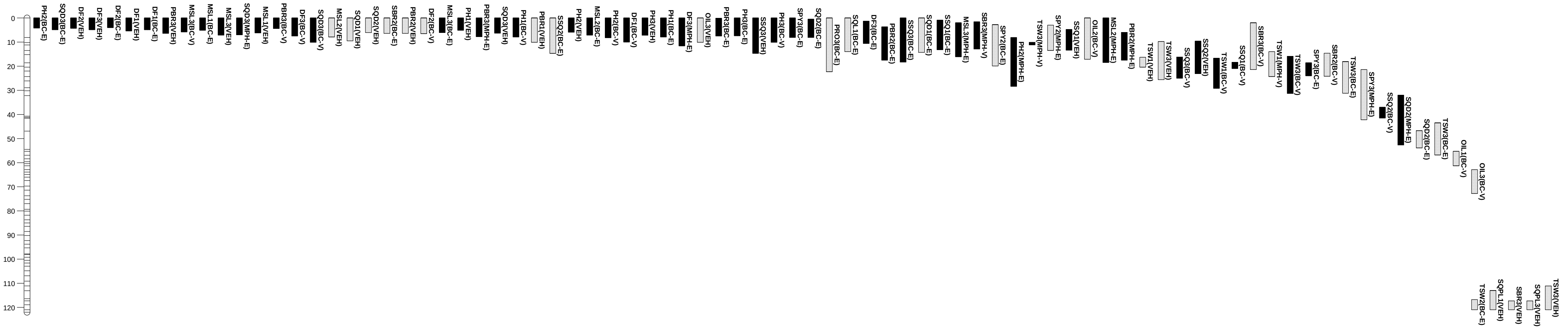

A8

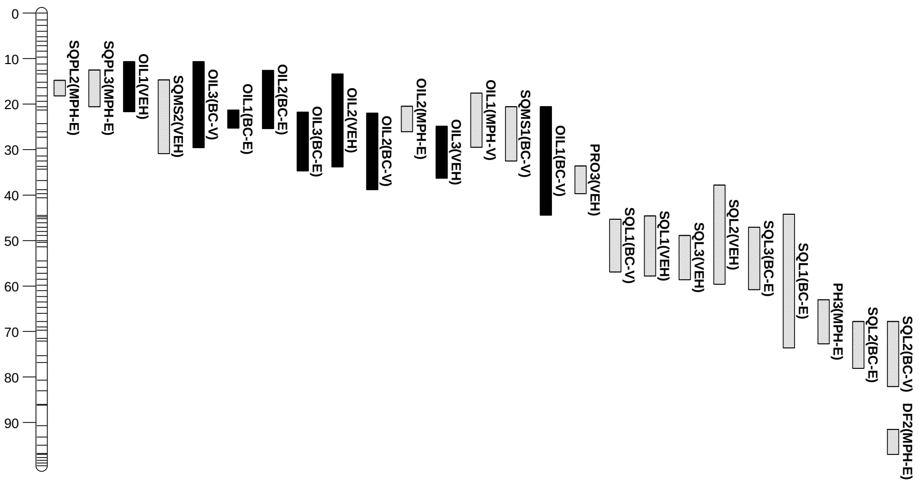
0
10
20
30
40
50
60
70
80
90
SQPL2(MPH-E)
SQPL3(MPH-E)
OIL1(VEH)
SQMS2(VEH)
OIL3(BC-V)
OIL1(BC-E)
OIL2(BC-E)
OIL3(BC-E)
OIL2(VEH)
OIL2(BC-V)
OIL2(MPH-E)
OIL3(VEH)
OIL1(MPH-V)
SQMS1(BC-V)
OIL1(BC-V)
PRO3(VEH)
SQL1(BC-V)
SQL1(VEH)
SQL3(VEH)
SQL2(VEH)
SQL3(BC-E)
SQL1(BC-E)
PH3(MPH-E)
SQL2(BC-E)
SQL2(BC-V)
DF2(MPH-E)

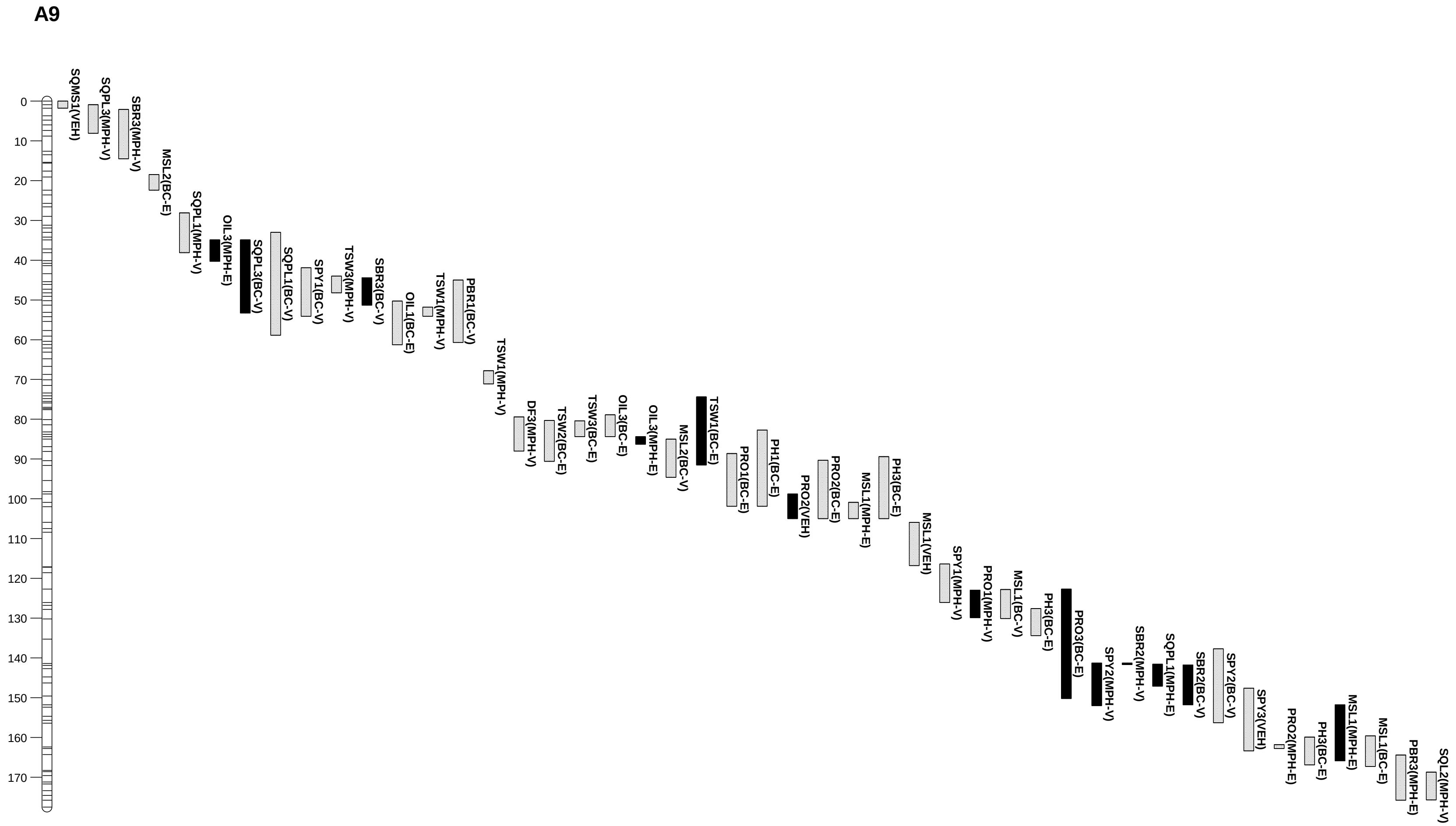
A9
SQMS1(VEH)
SQPL3(MPH-V)
SBR3(MPH-V)
MSL2(BC-E)
SQPL1(MPH-V)
OIL3(MPH-E)
SQPL3(BC-V)
SQPL1(BC-V)
SPY1(BC-V)
TSW3(MPH-V)
SBR3(BC-V)
OIL1(BC-E)
TSW1(MPH-V)
PBR1(BC-V)
TSW1(MPH-V)
DF3(MPH-V)
TSW2(BC-E)
TSW3(BC-E)
OIL3(BC-E)
OIL3(MPH-E)
MSL2(BC-V)
TSW1(BC-E)
PRO1(BC-E)
PH1(BC-E)
PRO2(VEH)
PRO2(BC-E)
MSL1(MPH-E)
PH3(BC-E)
MSL1(VEH)
SPY1(MPH-V)
PRO1(MPH-V)
MSL1(BC-V)
PH3(BC-E)
PRO3(BC-E)
SPY2(MPH-V)
SBR2(MPH-V)
SQPL1(MPH-E)
SBR2(BC-V)
SPY2(BC-V)
SPY3(VEH)
PRO2(MPH-E)
PH3(BC-E)
MSL1(MPH-E)
MSL1(BC-E)
PBR3(MPH-E)
SQL2(MPH-V)

A10

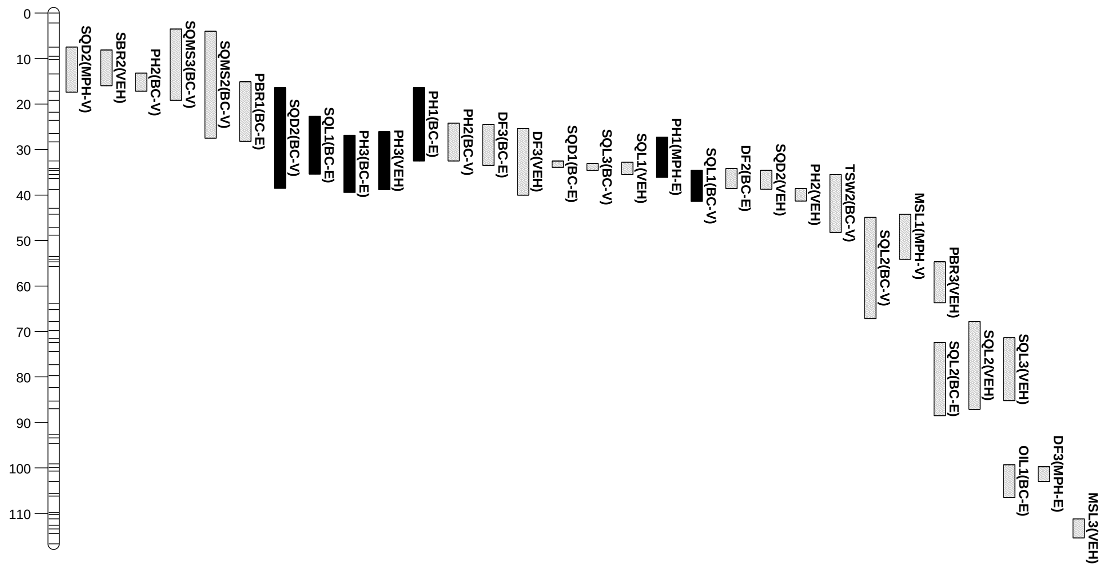
0
10
20
30
40
50
60
70
80
90
100
110
SQD2(MPH-V)
SBR2(VEH)
PH2(BC-V)
SQMS3(BC-V)
SQMS2(BC-V)
PBR1(BC-E)
SQD2(BC-V)
SQL1(BC-E)
PH3(BC-E)
PH3(VEH)
PH1(BC-E)
PH2(BC-V)
DF3(BC-E)
DF3(VEH)
SQD1(BC-E)
SQL3(BC-V)
SQL1(VEH)
PH1(MPH-E)
SQL1(BC-V)
DF2(BC-E)
SQD2(VEH)
PH2(VEH)
TSW2(BC-V)
SQL2(BC-V)
MSL1(MPH-V)
PBR3(VEH)
SQL2(BC-E)
SQL2(VEH)
SQL3(VEH)
OIL1(BC-E)
DF3(MPH-E)
MSL3(VEH)

B1

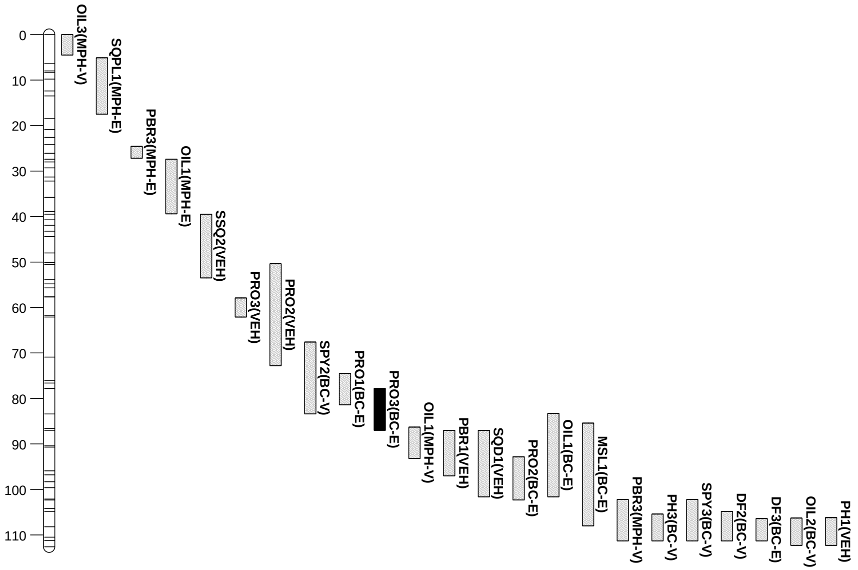
0
10
20
30
40
50
60
70
80
90
100
110
OIL3(MPH-V)
SQPL1(MPH-E)
PBR3(MPH-E)
OIL1(MPH-E)
SSQ2(VEH)
PRO3(VEH)
PRO2(VEH)
SPY2(BC-V)
PRO1(BC-E)
PRO3(BC-E)
OIL1(MPH-V)
PBR1(VEH)
SQD1(VEH)
PRO2(BC-E)
OIL1(BC-E)
MSL1(BC-E)
PBR3(MPH-V)
PH3(BC-V)
SPY3(BC-V)
DF2(BC-V)
DF3(BC-E)
OIL2(BC-V)
PH1(VEH)

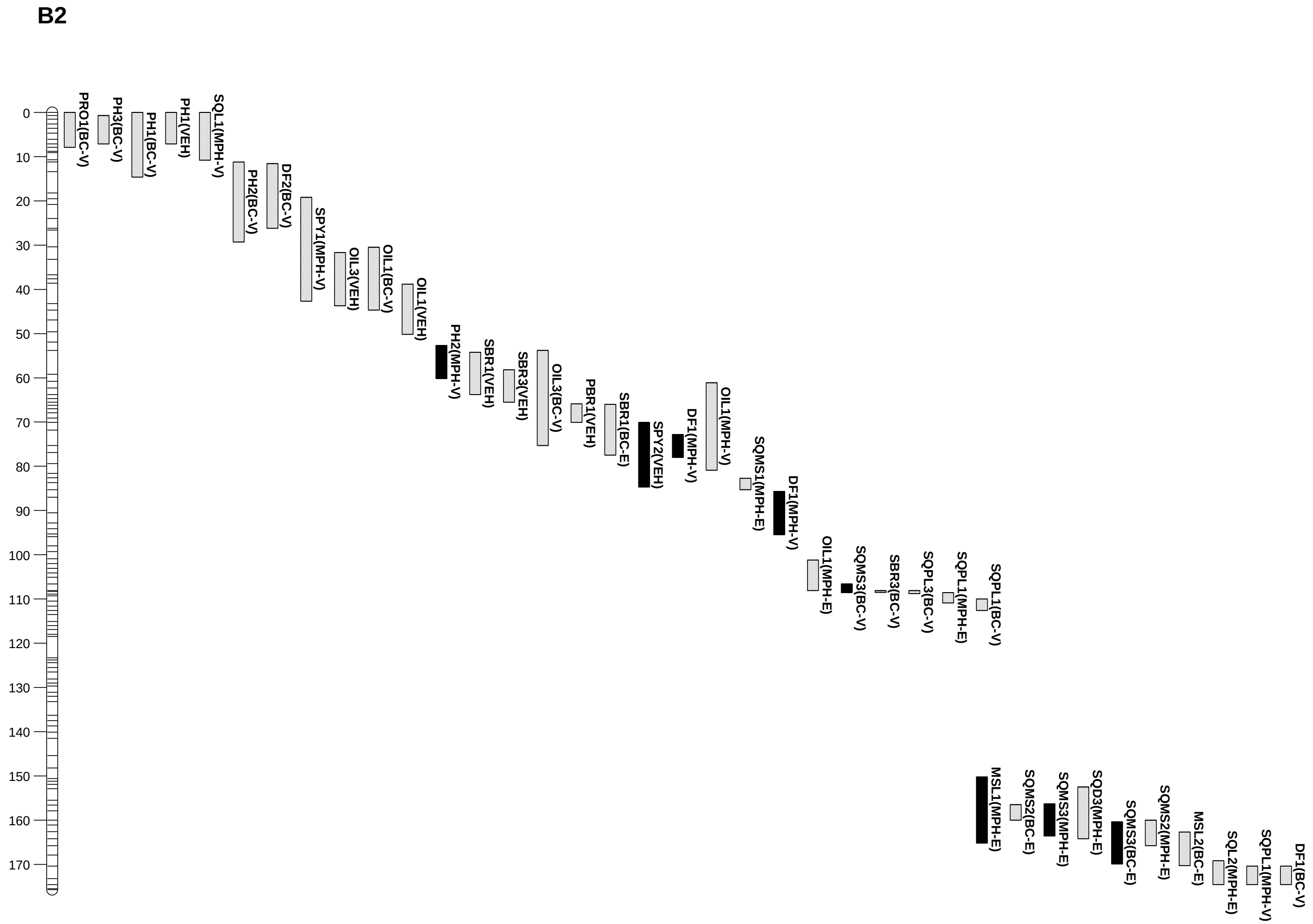
B2
PRO1(BC-V)
PH3(BC-V)
PH1(BC-V)
PH1(VEH)
SQL1(MPH-V)
PH2(BC-V)
DF2(BC-V)
SPY1(MPH-V)
OIL3(VEH)
OIL1(BC-V)
OIL1(VEH)
PH2(MPH-V)
SBR1(VEH)
SBR3(VEH)
OIL3(BC-V)
PBR1(VEH)
SBR1(BC-E)
SPY2(VEH)
DF1(MPH-V)
OIL1(MPH-V)
SQMS1(MPH-E)
DF1(MPH-V)
OIL1(MPH-E)
SQMS3(BC-V)
SBR3(BC-V)
SQPL3(BC-V)
SQPL1(MPH-E)
SQPL1(BC-V)
MSL1(MPH-E)
SQMS2(BC-E)
SQMS3(MPH-E)
SQD3(MPH-E)
SQMS3(BC-E)
SQMS2(MPH-E)
MSL2(BC-E)
SQL2(MPH-E)
SQPL1(MPH-V)
DF1(BC-V)

B3

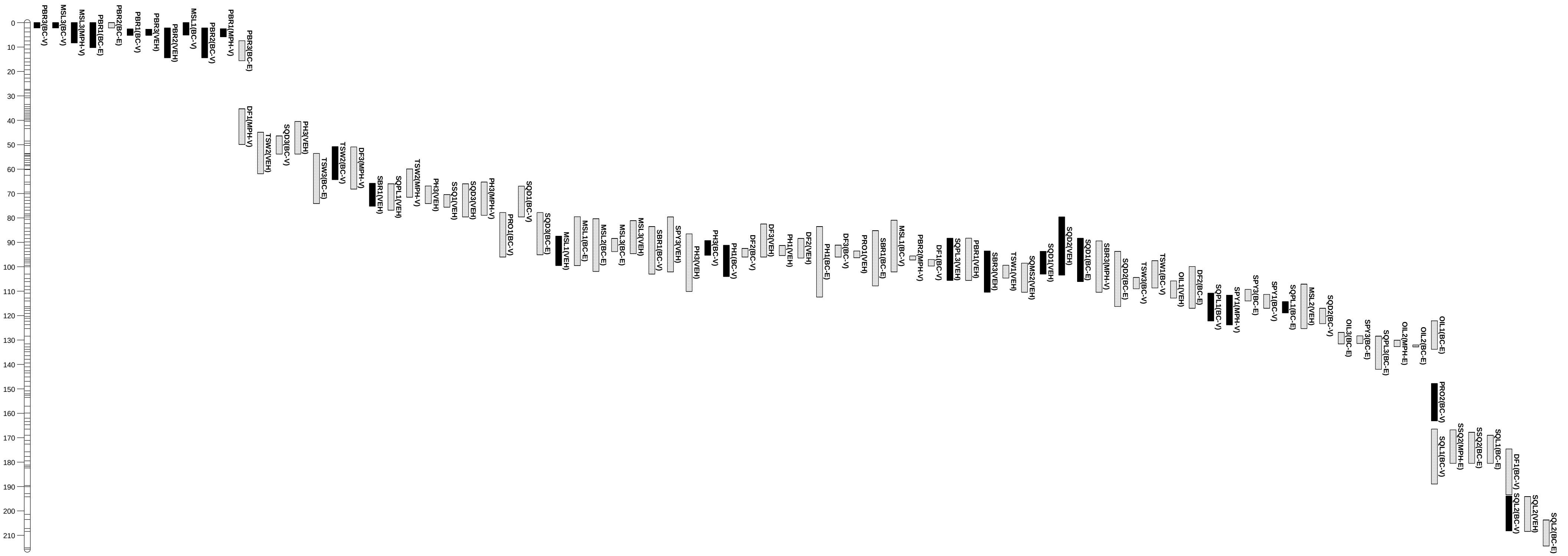

B4

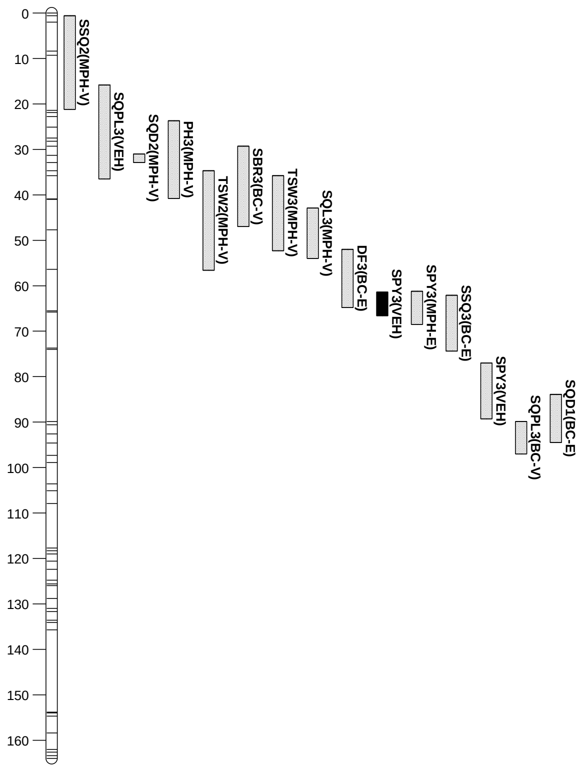
0
10
20
30
40
50
60
70
80
90
100
110
120
130
140
150
160
SSQ2(MPH-V)
SQPL3(VEH)
SQD2(MPH-V)
PH3(MPH-V)
TSW2(MPH-V)
SBR3(BC-V)
TSW3(MPH-V)
SQL3(MPH-V)
DF3(BC-E)
SPY3(VEH)
SPY3(MPH-E)
SSQ3(BC-E)
SPY3(VEH)
SQPL3(BC-V)
SQD1(BC-E)

B5

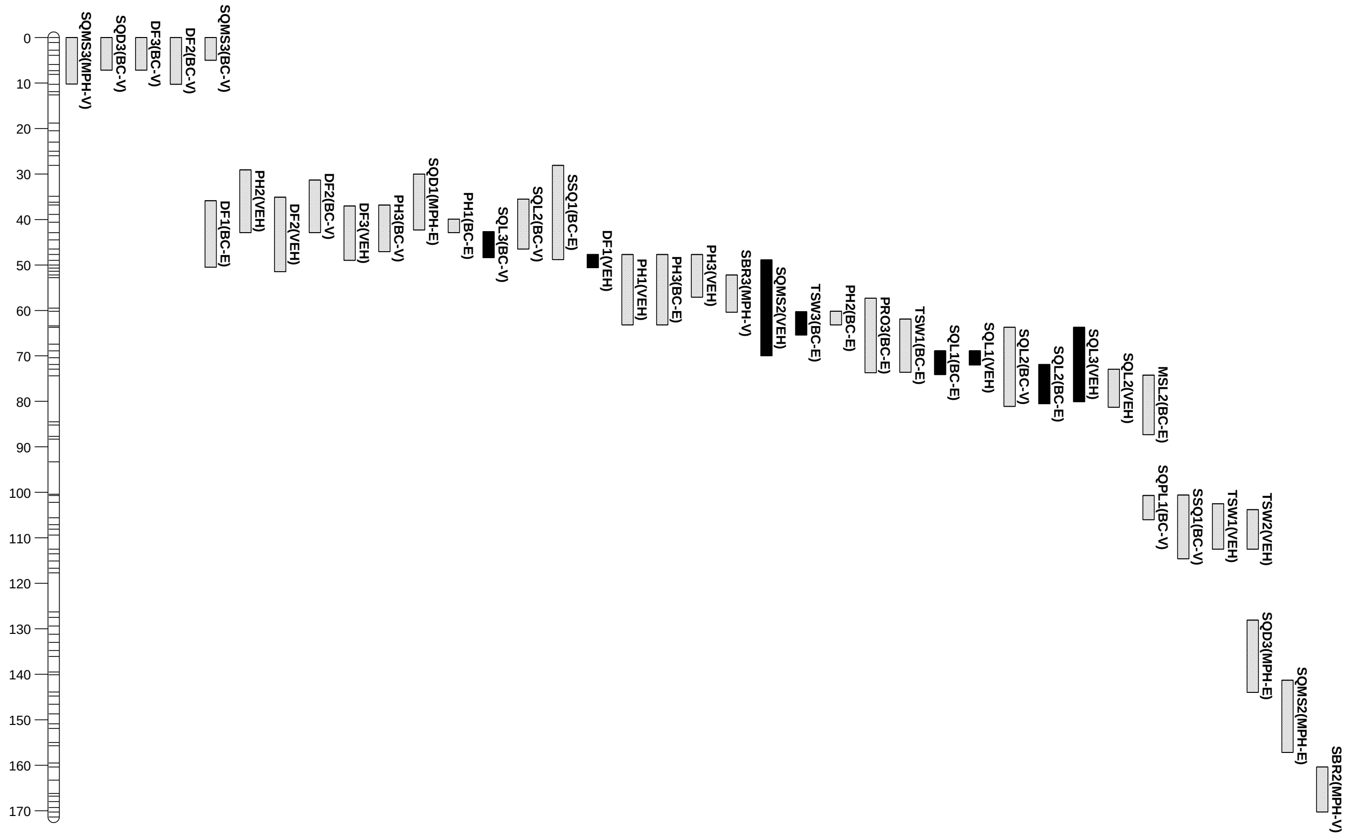

0
10
20
30
40
50
60
70
80
90
100
110
120
130
140
150
160
170
SQMS3(MPH-V)
SQD3(BC-V)
DF3(BC-V)
DF2(BC-V)
SQMS3(BC-V)
DF1(BC-E)
PH2(VEH)
DF2(VEH)
DF2(BC-V)
DF3(VEH)
PH3(BC-V)
SQD1(MPH-E)
PH1(BC-E)
SQL3(BC-V)
SQL2(BC-V)
SSQ1(BC-E)
DF1(VEH)
PH1(VEH)
PH3(BC-E)
PH3(VEH)
SBR3(MPH-V)
SQMS2(VEH)
TSW3(BC-E)
PH2(BC-E)
PRO3(BC-E)
TSW1(BC-E)
SQL1(BC-E)
SQL1(VEH)
SQL2(BC-V)
SQL2(BC-E)
SQL3(VEH)
SQL2(VEH)
MSL2(BC-E)
SQPL1(BC-V)
SSQ1(BC-V)
TSW1(VEH)
TSW2(VEH)
SQD3(MPH-E)
SQMS2(MPH-E)
SBR2(MPH-V)

B6

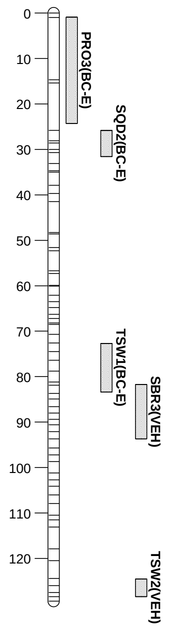
0
10
20
30
40
50
60
70
80
90
100
110
120
PRO3(BC-E)
SQD2(BC-E)
TSW1(BC-E)
SBR3(VEH)
TSW2(VEH)

B7

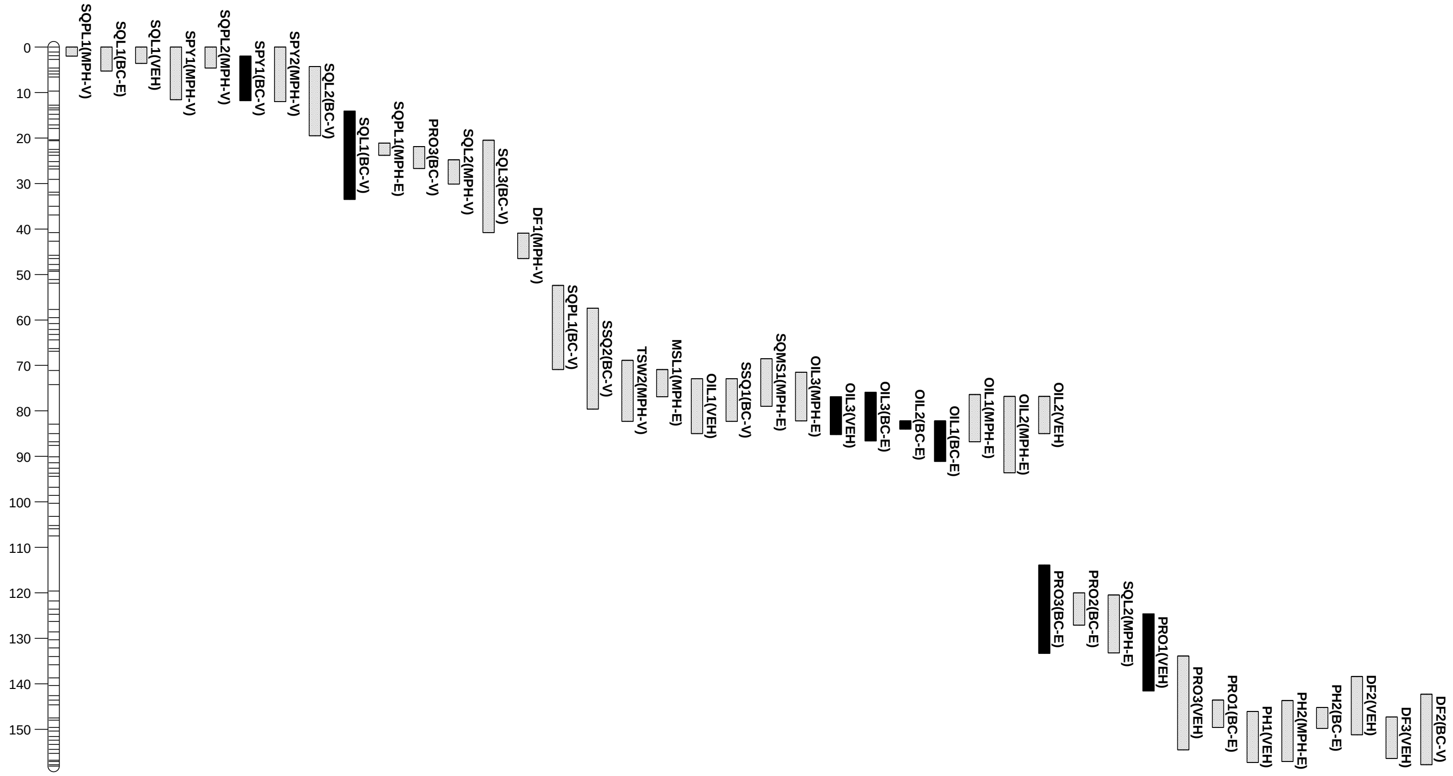
0
10
20
30
40
50
60
70
80
90
100
110
120
130
140
150
SQPL1(MPH-V)
SQL1(BC-E)
SQL1(VEH)
SPY1(MPH-V)
SQPL2(MPH-V)
SPY1(BC-V)
SPY2(MPH-V)
SQL2(BC-V)
SQL1(BC-V)
SQPL1(MPH-E)
PRO3(BC-V)
SQL2(MPH-V)
SQL3(BC-V)
DF1(MPH-V)
SQPL1(BC-V)
SSQ2(BC-V)
TSW2(MPH-V)
MSL1(MPH-E)
OIL1(VEH)
SSQ1(BC-V)
SQMS1(MPH-E)
OIL3(MPH-E)
OIL3(VEH)
OIL3(BC-E)
OIL2(BC-E)
OIL1(BC-E)
OIL1(MPH-E)
OIL2(MPH-E)
OIL2(VEH)
PRO3(BC-E)
PRO2(BC-E)
SQL2(MPH-E)
PRO1(VEH)
PRO3(VEH)
PRO1(BC-E)
PH1(VEH)
PH2(MPH-E)
PH2(BC-E)
DF2(VEH)
DF3(VEH)
DF2(BC-V)

B8

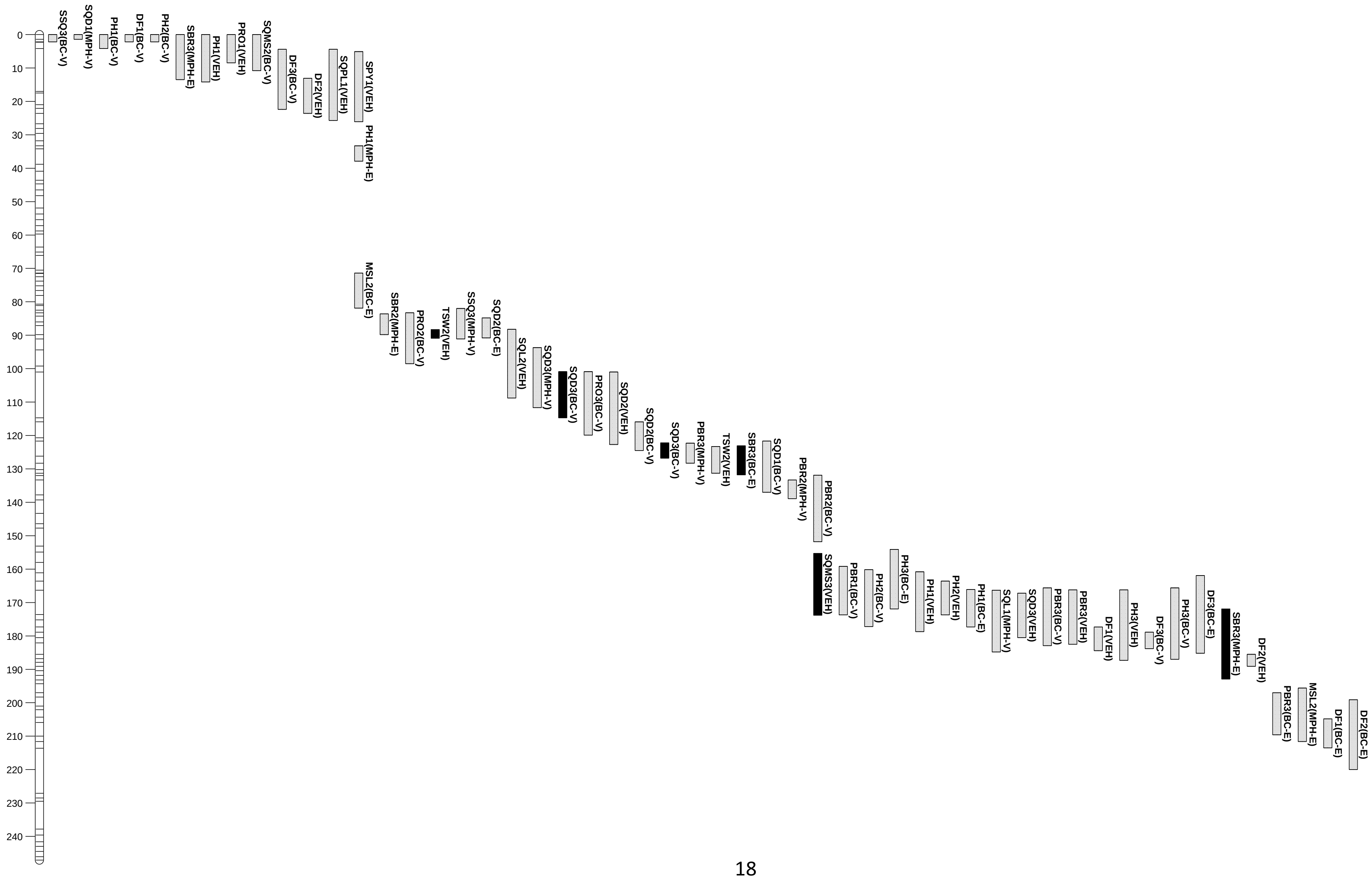
0
10
20
30
40
50
60
70
80
90
100
110
120
130
140
150
160
170
180
190
200
210
220
230
240
SSQ3(BC-V)
SQD1(MPH-V)
PH1(BC-V)
DF1(BC-V)
PH2(BC-V)
SBR3(MPH-E)
PH1(VEH)
PRO1(VEH)
SQMS2(BC-V)
DF3(BC-V)
DF2(VEH)
SQPL1(VEH)
SPY1(VEH)
PH1(MPH-E)
MSL2(BC-E)
SBR2(MPH-E)
PRO2(BC-V)
TSW2(VEH)
SSQ3(MPH-V)
SQD2(BC-E)
SQL2(VEH)
SQD3(MPH-V)
SQD3(BC-V)
PRO3(BC-V)
SQD2(VEH)
SQD2(BC-V)
SQD3(BC-V)
PBR3(MPH-V)
TSW2(VEH)
SBR3(BC-E)
SQD1(BC-V)
PBR2(MPH-V)
PBR2(BC-V)
SQMS3(VEH)
PBR1(BC-V)
PH2(BC-V)
PH3(BC-E)
PH1(VEH)
PH2(VEH)
PH1(BC-E)
SQL1(MPH-V)
SQD3(VEH)
PBR3(BC-V)
PBR3(VEH)
DF1(VEH)
PH3(VEH)
DF3(BC-V)
PH3(BC-V)
DF3(BC-E)
SBR3(MPH-E)
DF2(VEH)
PBR3(BC-E)
MSL2(MPH-E)
DF1(BC-E)
DF2(BC-E)
